# A Maximal Element Theorem in *FWC*-Spaces and Its Applications

**DOI:** 10.1155/2014/890696

**Published:** 2014-03-20

**Authors:** Haishu Lu, Qingwen Hu, Yulin Miao

**Affiliations:** ^1^School of Business, Jiangsu University of Technology, Changzhou, Jiangsu 213001, China; ^2^Department of Mathematical Sciences, University of Texas at Dallas, Richardson, TX 75080, USA

## Abstract

A maximal element theorem is proved in finite weakly convex spaces (*FWC*-spaces, in short) which have no linear, convex, and topological structure. Using the maximal element theorem, we develop new existence theorems of solutions to variational relation problem, generalized equilibrium problem, equilibrium problem with lower and upper bounds, and minimax problem in *FWC*-spaces. The results represented in this paper unify and extend some known results in the literature.

## 1. Introduction and Preliminaries

In 1983, by using fixed point theorems for set-valued mappings, Yannelis and Prabhakar [1] proved three existence theorems of maximal elements under the setting of locally convex topological vector spaces. In 1985, Yannelis [2] improved the Fan-Browder-type fixed point theorem and obtained an existence result of maximal elements by using this fixed point theorem. Since then, many maximal element theorems and their applications have been established in the setting of topological vector spaces; see, for example, [3–8] and the references therein.

It is well known that the linearity and convexity assumptions play crucial roles in most of the known existence results of maximal elements, which strictly restrict the applicable range of these maximal element theorems. Considering this fact, Zhang and Wu [9] proved an existence theorem of maximal elements in noncompact *H*-spaces and obtained some minimax inequalities, variational inequalities, and quasivariational inequalities by using this maximal element theorem. Subsequently, Wu [10] used existence theorems of maximal elements to prove equilibrium existence theorems for qualitative games and abstract economies in noncompact *H*-spaces. Recently, by using a generalization of the Fan-Browder fixed point theorem, Balaj and Lin [11] proved a new fixed point theorem for set-valued mappings in *G*-convex spaces from which they derived several coincidence theorems and existence theorems for maximal elements. As applications, they obtained some existence theorems of solutions to the generalized equilibrium problem and minimax problem.

Motivated and inspired by the work mentioned above, in this paper, we prove a new maximal element theorem in *FWC*-spaces (see Definition 1) without any linear, convex, and topological structure. As applications of this theorem, we obtain some new existence theorems of solutions to variational relation problem, generalized equilibrium problem, equilibrium problem with lower and upper bounds, and minimax problem in *FWC*-spaces.

Now, we introduce some notation and definitions. For a nonempty set *X*, 2^*X*^ and 〈*X*〉 denote the family of all subsets of *X* and the family of nonempty finite subsets of *X*, respectively. For every *A* ∈ 〈*X*〉, |*A*| denotes the cardinality of *A*. If *X* is a topological space, then $\bar{A}$ denotes the closure of *A*⊆*X*. If *X* is a vector space, then we denote by co⁡*A* the convex hull of *A*⊆*X*. Let *T* : *X* → 2^*Y*^ be a set-valued mapping with *Y* being a nonempty set. We define the mapping *T* ^−1^ : *Y* → 2^*X*^ by *T* ^−1^(*y*) = {*x* ∈ *X* : *y* ∈ *T*(*x*)} for each *y* ∈ *Y*. If *Y* is a topological space, we say that *T* : *X* → 2^*Y*^ is compact if $\bar{T(X)}\subseteq Y$ is compact. If *X* and *Y* are both topological spaces, we say that *T* : *X* → 2^*Y*^ is upper semicontinuous (resp., lower semicontinuous) if for every closed subset *B* of *Y*, the set {*x* ∈ *X* : *T*(*x*)⋂*B* ≠ *∅*} (resp., {*x* ∈ *X* : *T*(*x*)⊆*B*}) is closed. Let Δ_*n*_ denote the standard *n*-dimensional simplex with vertices {*e*_0_, *e*_1_,…, *e*_*n*_}. For a nonempty subset *J*⊆{0,1,…, *n*}, let Δ_|*J*|−1_ denote the convex hull of the vertices {*e*_*j*_ : *j* ∈ *J*}.

A nonempty topological space *X* is contractible if the identity mapping on *X* is homotopic to a constant mapping. Every nonempty convex subset of a topological vector space is contractible, but the converse is not true in general. A subset *A* of a topological space *X* is called to be compactly closed (resp., compactly open) in *X* if for each nonempty compact subset *C* of *X*, *A*∩*C* is closed (resp., open) in *C*. The notions of compactly closed (resp., compactly open) sets are true generalizations of closed (resp., open) sets. Note that there exists a nonempty subset *A* of the topological vector space ℝ^ℝ^ such that for each nonempty compact subset *C* of ℝ^ℝ^, *A*∩*C* is closed in *C*, but *A* is not closed. For details, see Kelley [12, page 240] or Wilansky [13, page 143].

Let *X* be a topological vector space. For every *N* = {*x*_0_, *x*_1_,…, *x*_*n*_}∈〈*X*〉, let us define a continuous mapping *φ* : Δ_*n*_ → *X* by *φ*(*p*) = ∑_*i*=0_^*n*^ *t*_*i*_ *x*_*i*_ for each *p* = ∑_*i*=0_^*n*^ *t*_*i*_ *e*_*i*_ ∈ Δ_*n*_. This mapping motivates us to introduce an abstract convex space which does not possess any linear, convex, and topological structure and is described in the following definition.

**Definition 1 (see [14])**

A triple (*X*, *D*; *φ*_*N*_) is said to be a finite weakly convex space (*FWC*-space, in short) if *X*, *D* are two nonempty sets and for each *N* = {*u*_0_, *u*_1_,…, *u*_*n*_}∈〈*D*〉 where some elements in *N* may be same, there exists a set-valued mapping *φ*_*N*_ : Δ_*n*_ → 2^*X*^ with nonempty values. When *D*⊆*X*, the space is denoted by (*X*⊇*D*; *φ*_*N*_). In case *X* = *D*, let (*X*; *φ*_*N*_): = (*X*, *X*; *φ*_*N*_). Let *A*⊆*D* and *B*⊆*X*. *B* is said to be an *FWC*-subspace of (*X*, *D*; *φ*_*N*_) relative to *A* if for each *N* = {*u*_0_,…, *u*_*n*_}∈〈*D*〉 and for each {*u*_*i*_0__, *u*_*i*_1__,…, *u*_*i*_*k*__}⊆*A*∩{*u*_0_, *u*_1_,…, *u*_*n*_}, we have *φ*_*N*_(Δ_*k*_)⊆*B*, where Δ_*k*_ = co⁡({*e*_*i*_0__, *e*_*i*_1__,…, *e*_*i*_*k*__}). We note that if *A* is nonempty and *B* is an *FWC*-subspace of (*X*, *D*; *φ*_*N*_) relative to *A*, then *B* is automatically nonempty. When *A* = *B*, *B* is said to be an *FWC*-subspace of (*X*⊇*D*; *φ*_*N*_).

It is worthwhile noticing that *X* and *D* in Definition 1 do not possess any linear, convex, and topological structure. Major examples of *FWC*-spaces are convex subsets of topological vector spaces, hyperconvex metric spaces introduced by Aronszajn and Panitchpakdi [15], Lassonde's convex spaces in [16], *H*-spaces introduced by Horvath [17], *G*-convex spaces introduced by Park and Kim [18], *L*-convex spaces introduced by Ben-El-Mechaiekh et al. [19], *G*-*H*-spaces introduced by Verma [20–22], pseudo-*H*-spaces introduced by Lai et al. [23], *GFC*-spaces due to Khanh et al. [24], *FC*-spaces due to Ding [25], and many other topological spaces with abstract convex structure (see, e.g., [26] and the references therein).

Taking Lassonde's convex space, *H*-space, and hyperconvex metric space as examples, we show that these three spaces are particular forms of *FWC*-spaces. Let *X* be a convex space in [16]; that is, a nonempty convex set in a vector space with any topology that induces the Euclidean topology on the convex hulls of its finite subsets. Then for every *N* = {*x*_0_, *x*_1_,…, *x*_*n*_}∈〈*X*〉, define a continuous mapping *φ*_*N*_ : Δ_*n*_ → co⁡(*N*)⊆*X* as follows:

(1) φN(∑j=0ntjej)=∑j=0ntjxjfor  each  (t0,t1,…,tn)=∑j=0ntjej∈Δn.

Therefore, (*X*; *φ*_*N*_) forms an *FWC*-space. Let (*X*; Γ_*N*_) be an *H*-space in [17], where {Γ_*N*_}_*N*∈〈*X*〉_ is a family of nonempty contractible subsets of *X* indexed by *N* ∈ 〈*X*〉 such that Γ_*N*_⊆Γ_*N*_′ whenever *N*⊆*N*′. Then by Theorem 1 of Horvath [17], for every *N* = {*x*_0_, *x*_1_,…, *x*_*n*_}∈〈*X*〉, there exists a continuous mapping *φ*_*N*_ : Δ_*n*_ → *X* and thus, (*X*; *φ*_*N*_) is an *FWC*-space. Let (*X*, *d*) be a hyperconvex metric space in [15]. Then by Lemma 2.3 of Yuan [27], we know that (*X*, *d*) is an *H*-space and thus, (*X*, *d*) is an *FWC*-space.

**Definition 2**

Let (*X*, *D*; *φ*_*N*_) be an *FWC*-space and *Y* a topological space. The class $\tilde{ℬ}(X,D,Y)$ of better admissible mappings is defined as follows: a set-valued mapping *T* : *X* → 2^*Y*^ belongs to $\tilde{ℬ}(X,D,Y)$ if and only if for every *N* = {*u*_0_, *u*_1_,…, *u*_*n*_}∈〈*D*〉 and for every continuous mapping *ψ* : *T*(*φ*_*N*_(Δ_*n*_)) → Δ_*n*_, the composition *ψ*∘*T*|_*φ*_*N*_(Δ_*n*_)_∘*φ*_*N*_ : Δ_*n*_ → 2^Δ_*n*_^ has a fixed point. When *X* = *D*, we will write $\tilde{ℬ}(X,Y)$ instead of $\tilde{ℬ}(X,D,Y)$.

**Remark 3**

Since *X* and *D* in Definition 2 are nonempty sets which do not possess any linear, convex, and topological structure, the class $\tilde{ℬ}(X,D,Y)$ includes many important classes of mappings as special cases, for example, the class of Kakutani's mappings *𝒦*(*X*, *Y*) (i.e., the upper semicontinuous set-valued mappings with nonempty compact convex values and codomain *Y* being convex set in a topological vector space), the class *𝒰*_*C*_^*K*^(*X*, *Y*) in Park and Kim [18], the class *𝒜*(*X*, *Y*) in Ben-El-Mechaiekh et al. [19], and the class *ℬ*(*X*, *Y*) in Ding [25].

**Example 4**

Let *D* = [0,1] and (*X*, *τ*) = ([0,1], {[0,1], *∅*, (0,1], [0, 1/2]}), where *τ* is a family of subsets of *X*. We can verify that (*X*, *τ*) is not a topological space. For simplicity, we write *X* instead of (*X*, *τ*). Let *Y* = (0,1] with the Euclidean metric topology. For each *N* = {*u*_0_, *u*_1_,…, *u*_*n*_}∈〈*D*〉, define a set-valued mapping *φ*_*N*_ : Δ_*n*_ → 2^*X*^ by *φ*_*N*_(*z*) = [∑_*i*=0_^*n*^ *λ*_*i*_ *d*_*i*_, 1] for each *z* = ∑_*i*=0_^*n*^ *λ*_*i*_ *e*_*i*_ ∈ Δ_*n*_. It is easy to see that (*X*, *D*; {*φ*_*N*_}) forms an *FWC*-space. Now we define a set-valued mapping *T* : *X* → 2^*Y*^ by

(2) $$\begin{array}{c} T\left(x\right)=\{\begin{array}{cc} \left[\frac{7}{10},\frac{4}{5}\right], & \text{if}\;\;x=1,\\ \left(\frac{3}{4},\frac{4}{5}\right], & \text{if}\;\;x\in \left[0,1\right). \end{array} \end{array}$$

Then for each *N* = {*u*_0_, *u*_1_,…, *u*_*n*_}∈〈*D*〉, we have *T*(*φ*_*N*_(Δ_*n*_)) = [7/10, 4/5]. Therefore, the composition *T*|_*φ*_*N*__∘*φ*_*N*_ : Δ_*n*_ → 2^*T*(*φ*_*N*_(Δ_*n*_))^ is an upper semicontinuous set-valued mapping with nonempty compact contractible values. By Lemma 1 of [28], for every continuous function *ψ* : *T*(*φ*_*N*_(Δ_*n*_)) → Δ_*n*_, the composition *ψ* ∘ *T*|_*φ*_*N*__ ∘ *φ*_*N*_ : Δ_*n*_ → 2^Δ_*n*_^ has a fixed point. Therefore, $T\in \tilde{ℬ}(X,Y)$.

**Lemma 5**

Let *I* be an index set. For each *i* ∈ *I*, let (*X*_*i*_, *D*_*i*_; *φ*_*N*_*i*__^*i*^) be an *FWC*-space. Let *X* = ∏_*i*∈*I*_ *X*_*i*_, *D* = ∏_*i*∈*I*_ *D*_*i*_, and *φ*_*N*_ = ∏_*i*∈*I*_ *φ*_*N*_*i*__^*i*^. Then (*X*, *D*; *φ*_*N*_) is also an *FWC*-space.

**Proof**

For each *i* ∈ *I*, let *π*_*i*_ : *X* → *X*_*i*_ be the projection of *X* onto *X*_*i*_. For every *N* = {*u*_0_, *u*_1_,…, *u*_*n*_}∈〈*D*〉, let *N*_*i*_ = *π*_*i*_(*N*) = {*π*_*i*_(*u*_0_), *π*_*i*_(*u*_1_),…, *π*_*i*_(*u*_*n*_)}. Since each (*X*_*i*_, *D*_*i*_; *φ*_*N*_*i*__) is an *FWC*-space, it follows that there exists a set-valued mapping *φ*_*N*_*i*__^*i*^ : Δ_*n*_ → 2^*X*_*i*_^ with nonempty values. Define a set-valued mapping *φ*_*N*_ : Δ_*n*_ → 2^*X*^ by

(3) $$\begin{array}{c} \varphi_{N}\left(z\right)=\prod_{i\in I}\varphi_{N_{i}}^{i}\left(z\right)\;\text{for}\;\;\text{each}\;\;z\in \Delta_{n}. \end{array}$$

It is clear that *φ*_*N*_ has nonempty values. Therefore, (*X*, *D*; *φ*_*N*_) is also an *FWC*-space.

## 2. A Maximal Element Theorem

Our first result is the following maximal element theorem.

**Theorem 6**

Let (*X*, *D*; *φ*_*N*_) be an FWC-space, *Y* a Hausdorff topological space, and *K* a nonempty compact subset of *Y*. Let *P* : *X* → 2^*Y*^, *H* : *Y* → 2^*D*^, *Q* : *D* → 2^*X*^, and $T\in \tilde{ℬ}(X,D,Y)$ be set-valued mappings such that

(i) for each *x* ∈ *X*, *T*(*x*)⊆*P*(*x*);
(ii) for each *u* ∈ *D*, *H* ^−1^(*u*) is compactly open;
(iii) for each *N* = {*u*_0_, *u*_1_,…, *u*_*n*_}∈〈*D*〉 and each {*u*_*i*_0__, *u*_*i*_1__,…, *u*_*i*_*k*__}⊆*N*,
  (4) $$\begin{array}{c} P\left(\varphi_{N}\left(\Delta_{k}\right)\right)⋂\left(\overset{k}{\underset{j=0}{⋂}}H^{-1}\left(u_{i_{j}}\right)\right)=\emptyset ; \end{array}$$
(iv) one of the following conditions holds:
  (iv_1_) there exists *N*_0_ ∈ 〈*D*〉 such that $\bar{T(X)}\setminus K\subseteq {⋃}_{u\in N_{0}}H^{-1}(u)$ and for each *N* = {*u*_0_, *u*_1_,…, *u*_*n*_}∈〈*D*〉, $\bar{T(\varphi_{N}(\Delta_{n}))}$ is a compact subset of *Y*;
  (iv_2_) for each *N* ∈ 〈*D*〉, there exists a subset *L*_*N*_ of *D* containing *N* such that *Q*(*L*_*N*_) is an *FWC*-subspace of (*X*, *D*; *φ*_*N*_) relative to *L*_*N*_, $\bar{\left(T\circ Q)(L_{N}\right)}$ is a compact subset of *Y*, and
    (5) $$\begin{array}{c} \bar{\left(T\circ Q)(L_{N}\right)}\setminus K\subseteq \underset{u\in L_{N}}{⋃}H^{-1}\left(u\right). \end{array}$$

Then there exists $\hat{y}\in \bar{T(X)}{⋂}_{}K$ such that $H(\hat{y})=\emptyset$.

**Proof**

We prove Theorem 6 distinguishing the following two cases.

*Case* (iv_1_). Assume (iv_1_) holds. Suppose that the conclusion of Theorem 6 does not hold. Then for each $y\in \bar{T(X)}{⋂}_{}K$, *H*(*y*) ≠ *∅* and hence, there exists $\bar{u}\in H(y)$; that is, $y\in H^{-1}(\bar{u})$. Therefore, we have

(6) $$\begin{array}{c} \bar{T(X)}⋂K\subseteq \underset{u\in D}{⋃}H^{-1}\left(u\right), \end{array}$$

which implies that $\bar{T(X)}{⋂}_{}K={⋃}_{u\in D}(H^{-1}(u){⋂}_{}\bar{T(X)}{⋂}_{}K)$. Since $\bar{T(X)}{⋂}_{}K$ is compact and each *H* ^−1^(*u*) is compactly open by (ii), it follows that there exists *N*_1_ ∈ 〈*D*〉 such that

(7) T(X)¯⋂K=⋃u∈N1(H−1(u)⋂T(X)¯⋂K)⊆⋃u∈N1H−1(u).

By the first part of (iv_1_), we have

(8) $$\begin{array}{c} \bar{T(X)}\setminus K\subseteq \underset{u\in N_{0}}{⋃}H^{-1}\left(u\right)\;\text{for}\;\;\text{some}\;\;N_{0}\in \langle D\rangle . \end{array}$$

Then it follows from (7) and (8) that

(9) $$\begin{array}{c} \bar{T(X)}=\left(\bar{T(X)}\setminus K\right)⋃\left(\bar{T(X)}⋂K\right)\subseteq \underset{u\in N}{⋃}H^{-1}\left(u\right), \end{array}$$

where *N* = *N*_0_⋃*N*_1_ = {*u*_0_, *u*_1_,…, *u*_*n*_}∈〈*D*〉. By the definition of *FWC*-spaces, there exists a set-valued mapping *φ*_*N*_ : Δ_*n*_ → 2^*X*^ with nonempty values. By the second part of (iv_1_), $\bar{T(\varphi_{N}(\Delta_{n}))}$ is compact subset of *Y*. By (9), we have

(10) $$\begin{array}{c} \bar{T(\varphi_{N}(\Delta_{n}))}\subseteq \bar{T(X)}\subseteq \underset{u\in N}{⋃}H^{-1}\left(u\right), \end{array}$$

and thus, $\bar{T(\varphi_{N}(\Delta_{n}))}={⋃}_{u\in N}(H^{-1}(u){⋂}_{}\bar{T(\varphi_{N}(\Delta_{n}))})$; that is, $\left\{H^{-1}(u){⋂}_{}\bar{T(\varphi_{N}(\Delta_{n}))}:u\in N\right\}$ is an open cover of the compact set $\bar{T(\varphi_{N}(\Delta_{n}))}$. Let {*λ*_*i*_}_*i*=0_^*n*^ be the partition of unity subordinated to this cover and then define a mapping $\psi :\bar{T(\varphi_{N}(\Delta_{n}))}\to \Delta_{n}$ by *ψ*(*y*) = ∑_*i*=0_^*n*^ *λ*_*i*_(*y*)*e*_*i*_ for each $y\in \bar{T(\varphi_{N}(\Delta_{n}))}$. Clearly, *ψ* is continuous and for each $y\in \bar{T(\varphi_{N}(\Delta_{n}))}$, we have

(11) $$\begin{array}{c} \psi \left(y\right)=\Sigma_{j\in J(y)}\lambda_{j}\left(y\right)e_{j}\in \Delta_{|J(y)|-1}, \end{array}$$

where *J*(*y*)⊆{0,1,…, *n*} is defined by *J*(*y*) = {*j* ∈ {0,1,…, *n*} : *λ*_*j*_(*y*) > 0}. Then we have

(12) j∈J(y)⟺λj(y)>0⟺y∈H−1(uj)⋂T(φN(Δn))¯⟹uj∈H(y).

Define a set-valued mapping $f:\bar{T(\varphi_{N}(\Delta_{n}))}\to 2^{X}$ as follows:

(13) $$\begin{array}{c} f\left(y\right)=\varphi_{N}\left(\psi \left(y\right)\right)\;\text{for}\;\;\text{each}\;\;y\in \bar{T(\varphi_{N}(\Delta_{n}))}. \end{array}$$

Now, we show that for each *y* ∈ *Y*, *X*∖*T* ^−1^(*y*) is an *FWC*-subspace of (*X*, *D*; *φ*_*N*_) relative to *H*(*y*). In fact, let *N* = {*u*_0_, *u*_1_,…, *u*_*n*_}∈〈*D*〉 and {*u*_*i*_0__, *u*_*i*_1__,…, *u*_*i*_*k*__}⊆*N*⋂*H*(*y*). Then *y* ∈ ⋂_*j*=0_^*k*^ *H* ^−1^(*u*_*i*_*j*__). By (iii), *y* ∉ *P*(*φ*_*N*_(Δ_*k*_)); that is, *P* ^−1^(*y*)⋂*φ*_*N*_(Δ_*k*_) = *∅*. Therefore, we have *φ*_*N*_(Δ_*k*_)⊆*X*∖*P* ^−1^(*y*). By (i), we know that *φ*_*N*_(Δ_*k*_)⊆*X*∖*T* ^−1^(*y*), which implies that for each *y* ∈ *Y*, *X*∖*T* ^−1^(*y*) is an *FWC*-subspace of (*X*, *D*; *φ*_*N*_) relative to *H*(*y*). Hence, by (12) and (13), we have

(14) f(y)=φN(ψ(y))⊆φN(Δ|J(y)|−1)⊆X∖T−1(y)∀y∈T(φN(Δn))¯.

This shows that

(15) $$\begin{array}{c} x\notin T^{-1}\left(y\right)\;\forall y\in \bar{T(\varphi_{N}(\Delta_{n}))}\;\;\text{and}\;\;\text{all}\;\;x\in f\left(y\right). \end{array}$$

On the other hand, since $T\in \tilde{ℬ}(X,D,Y)$, it follows that the composition mapping *ψ*∘*T*|_*φ*_*N*_(Δ_*n*_)_∘*φ*_*N*_ has a fixed point *z*_0_ ∈ Δ_*n*_; that is, *z*_0_ ∈ *ψ*∘*T*|_*φ*_*N*_(Δ_*n*_)_∘*φ*_*N*_(*z*_0_). Let $\bar{y}\in T{|}_{\varphi_{N}(\Delta_{n})}(\varphi_{N}(z_{0}))$ such that $z_{0}=\psi (\bar{y})$. Choose $\bar{x}\in \varphi_{N}(z_{0})$ such that

(16) $$\begin{array}{c} \bar{y}\in T{|}_{\varphi_{N}(\Delta_{n})}\left(\bar{x}\right)\subseteq \bar{T(\varphi_{N}(\Delta_{n}))}. \end{array}$$

Then by (13) and (16), we have

(17) $$\begin{array}{c} \bar{x}\in \varphi_{N}\left(z_{0}\right)=\varphi_{N}\left(\psi \left(\bar{y}\right)\right)=f\left(\bar{y}\right),\;\bar{x}\in T^{-1}\left(\bar{y}\right), \end{array}$$

which contradicts (15). Thus, there must exist a point $\hat{y}\in K{⋂}_{}\bar{T(X)}$ such that $H(\hat{y})=\emptyset$.

*Case* (iv_2_). Assume (iv_2_) holds. Suppose that the conclusion of Theorem 6 is not true. Then by using the same method as in Case (iv_1_), we have

(18) $$\begin{array}{c} \bar{T(X)}⋂K\subseteq \underset{u\in D}{⋃}H^{-1}\left(u\right)=Y\setminus \underset{u\in D}{⋂}\left(Y\setminus H^{-1}\left(u\right)\right), \end{array}$$

which implies that ${⋂}_{u\in D}(K{⋂}_{}\bar{T(X)}{⋂}_{}(Y\setminus H^{-1}(u)))=\emptyset$. By (ii), we know that $\left\{K{⋂}_{}\bar{T(X)}{⋂}_{}(Y\setminus H^{-1}(u)):u\in D\right\}$ is a family of closed sets in *K*. Thus, there exists *N* ∈ 〈*D*〉 such that

(19) ∅=⋂u∈N(T(X)¯⋂K⋂(Y∖H−1(u)))=T(X)¯⋂K⋂(⋂u∈N(Y∖H−1(u)));

that is, $\bar{T(X)}{⋂}_{}({⋂}_{u\in N}(Y\setminus H^{-1}(u)))\subseteq Y\setminus K$. By (iv_2_), there exists a subset *L*_*N*_ of *D* containing *N* such that *Q*(*L*_*N*_) is an *FWC*-subspace of (*X*, *D*; *φ*_*N*_) relative to *L*_*N*_ and

(20) $$\begin{array}{c} \bar{\left(T\circ Q)(L_{N}\right)}\setminus K\subseteq \underset{u\in L_{N}}{⋃}H^{-1}\left(u\right). \end{array}$$

Therefore, we have

(21) (T∘Q)(LN)¯⋂(⋂u∈LN(Y∖H−1(u)))  =(T∘Q)(LN)¯⋂(Y∖⋃u∈LNH−1(u))  ⊆(T∘Q)(LN)¯⋂(Y∖((T∘Q)(LN)¯∖K))  ⊆K.

Since $\bar{\left(T\circ Q)(L_{N}\right)}{⋂}_{}({⋂}_{u\in L_{N}}(Y\setminus H^{-1}(u)))\subseteq \bar{T(X)}{⋂}_{}({⋂}_{u\in N}(Y\setminus H^{-1}(u)))\subseteq Y\setminus K$, it follows that $\bar{\left(T\circ Q)(L_{N}\right)}{⋂}_{}({⋂}_{u\in L_{N}}(Y\setminus H^{-1}(u)))=\emptyset$. Therefore, we have

(22) $$\begin{array}{c} \bar{\left(T\circ Q)(L_{N}\right)}\subseteq \underset{u\in L_{N}}{⋃}H^{-1}\left(u\right). \end{array}$$

Since $\bar{\left(T\circ Q)(L_{N}\right)}$ is a compact subset of *Y*, it follows from (ii) and (22) that there exists *M* = {*u*_0_, *u*_1_,…, *u*_*m*_}∈〈*L*_*N*_〉 such that

(23) $$\begin{array}{c} \bar{\left(T\circ Q)(L_{N}\right)}=\underset{u\in M}{⋃}\left(\bar{\left(T\circ Q\right)\left(L_{N}\right)}⋂H^{-1}\left(u\right)\right). \end{array}$$

By the fact that *Q*(*L*_*N*_) is an *FWC*-subspace of (*X*, *D*; *φ*_*N*_) relative to *L*_*N*_, we can see that the triple (*Q*(*L*_*N*_), *L*_*N*_; *φ*_*N*_) is also an *FWC*-space. Hence, there exists a set-valued mapping *φ*_*M*_ : Δ_*m*_ → 2^*Q*(*L*_*N*_)^ with nonempty values. Assume that {*β*_*i*_}_*i*=0_^*m*^ is the partition of unity subordinated to the open cover $\left\{\bar{\left(T\circ Q)(L_{N}\right)}{⋂}_{}H^{-1}(u_{i}):0\leq i\leq m\right\}$. Then for every *i* ∈ {0,1,…, *n*}, we have

(24) $$\begin{array}{c} \beta_{i}:\bar{\left(T\circ Q\right)\left(L_{N}\right)}⟶\left[0,1\right]\;\;\text{is}\;\;\text{continuous};\\ \beta_{i}\left(y\right)>0⟺y\in \bar{\left(T\circ Q)(L_{N}\right)}⋂H^{-1}\left(u_{i}\right);\\ \sum_{i=0}^{m}\beta_{i}\left(y\right)=1\;\text{for}\;\;\text{each}\;\;y\in \bar{\left(T\circ Q)(L_{N}\right)}. \end{array}$$

Furthermore, we define a continuous mapping $\psi :\bar{\left(T\circ Q)(L_{N}\right)}\to \Delta_{m}$ by *ψ*(*y*) = ∑_*i*=0_^*m*^ *β*_*i*_(*y*)*e*_*i*_ for each $y\in \bar{\left(T\circ Q)(L_{N}\right)}$. Let *T*′ : = *T*|_*Q*(*L*_*N*_)_. Since $T\in \tilde{ℬ}(X,D,Y)$, it follows that $T^{\prime }\in \tilde{ℬ}(Q(L_{N}),L_{N},Y)$. Then the composition *ψ*∘*T*′|_*φ*_*M*_(Δ_*m*_)_∘*φ*_*M*_ : Δ_*m*_ → 2^Δ_*m*_^ has a fixed point *z*_0_ ∈ Δ_*m*_; that is, *z*_0_ ∈ *ψ*∘*T*′|_*φ*_*M*_(Δ_*m*_)_∘*φ*_*M*_(*z*_0_). Let $\bar{y}\in T^{\prime }(\varphi_{M}(z_{0}))$ such that $z_{0}=\psi (\bar{y})$. Then we have

(25) $$\begin{array}{c} z_{0}=\psi \left(\bar{y}\right)=\sum_{j\in J(\bar{y})}\beta_{j}\left(\bar{y}\right)e_{j}\in \Delta_{|J(\bar{y})|-1}, \end{array}$$

where $J(\bar{y})=\{j\in \{0,1,\ldots ,m\}:\beta_{j}(\bar{y})\neq 0\}$. Let *H*′^−1^ : = *H* ^−1^|_*L*_*N*__. By (i) and (iii), for each *N* = {*u*_0_, *u*_1_,…, *u*_*m*_}∈〈*L*_*N*_〉 and each {*u*_*i*_0__, *u*_*i*_1__,…, *u*_*i*_*k*__}⊆*N*, we have

(26) $$\begin{array}{c} T^{\prime }\left(\varphi_{M}\left(\Delta_{k}\right)\right)⋂\left(\overset{k}{\underset{j=0}{⋂}}{H^{\prime }}^{-1}\left(u_{i_{j}}\right)\right)=\emptyset ; \end{array}$$

thus, we have the following:

(27) y¯∈T′(φM(ψ(y¯)))⊆T′(φM(Δ|J(y¯)|−1))⊆⋃j∈J(y¯)(Y∖H′−1(uj)).

Hence, there exists $j\in J(\bar{y})$ such that $\bar{y}\notin {H^{\prime }}^{-1}(u_{j})$. On the other hand, by the definitions of $J(\bar{y})$ and of the partition {*β*_*i*_}_*i*=0_^*m*^, we have

(28) y¯∈(T∘Q)(LN)¯⋂H−1(uj)⊆H−1(uj)=H′−1(uj),

which is a contradiction. Therefore, the conclusion of Theorem 6 holds.

**Remark 7**

(iii) of Theorem 6 can be replaced by the following equivalent condition:

(iii)′ for each *y* ∈ *Y*, *X*∖*P* ^−1^(*y*) is an *FWC*-subspace of (*X*, *D*; *φ*_*N*_) relative to *H*(*y*).

**Proof**

(iii) ⇒(iii)′: let *N* = {*u*_0_, *u*_1_,…, *u*_*n*_}∈〈*D*〉 and {*u*_*i*_0__, *u*_*i*_1__,…, *u*_*i*_*k*__}⊆*N*⋂*H*(*y*). Then {*u*_*i*_0__, *u*_*i*_1__,…, *u*_*i*_*k*__}⊆*H*(*y*). Thus, *y* ∉ ⋃_*j*=0_^*k*^ *Y*∖*H* ^−1^(*u*_*i*_*j*__). Since *P*(*φ*_*N*_(Δ_*k*_))⊆⋃_*j*=0_^*k*^(*Y*∖*H* ^−1^(*u*_*i*_*j*__)) by (iii) of Theorem 6, it follows that *y* ∉ *P*(*φ*_*N*_(Δ_*k*_)); that is, *P* ^−1^(*y*)⋂*φ*_*N*_(Δ_*k*_) = *∅*. Therefore, we have *φ*_*N*_(Δ_*k*_)⊆*X*∖*P* ^−1^(*y*), which implies that (iii)′ holds.

(iii)′⇒ (iii): let *N* = {*u*_0_, *u*_1_,…, *u*_*n*_}∈〈*D*〉, {*u*_*i*_0__, *u*_*i*_1__,…, *u*_*i*_*k*__}⊆*N*, and *y* ∈ *P*(*φ*_*N*_(Δ_*k*_)). Then there exists *x* ∈ *φ*_*N*_(Δ_*k*_) such that *y* ∈ *P*(*x*). Therefore, we get

(29) $$\begin{array}{c} x\in P^{-1}\left(y\right)⋂\varphi_{N}\left(\Delta_{k}\right)\neq \emptyset . \end{array}$$

This means that *φ*_*N*_(Δ_*k*_)⊈*X*∖*P* ^−1^(*y*). By (iii)′, we have {*u*_*i*_0__, *u*_*i*_1__,…, *u*_*i*_*k*__}⊈*N*⋂*H*(*y*) and hence, {*u*_*i*_0__, *u*_*i*_1__,…, *u*_*i*_*k*__}⋂(*D*∖*H*(*y*)) ≠ *∅*. Let *d* ∈ {*u*_*i*_0__, *u*_*i*_1__,…, *u*_*i*_*k*__}⋂(*D*∖*H*(*y*)). Then *y* ∈ *Y*∖*H* ^−1^(*d*)⊆⋃_*j*=0_^*k*^(*Y*∖*H* ^−1^(*u*_*i*_*j*__)), which implies that

(30) $$\begin{array}{c} P\left(\varphi_{N}\left(\Delta_{k}\right)\right)⋂\left(\overset{k}{\underset{j=0}{⋂}}H^{-1}\left(u_{i_{j}}\right)\right)=\emptyset . \end{array}$$

**Example 8**

Let *Y* = (−1, +*∞*) be endowed with the Euclidean topology. Let *D* = [−4,0] and (*X*, *τ*) = ([0,4], {[0,4], *∅*, [0,1], [1,2], [2,3], [3,4]}), where *τ* is a family of subsets of *X*. It is easy to check that (*X*, *τ*) is not a topological space. For simplicity, we will write *X* instead of (*X*, *τ*). Define a set-valued mappings *H* : *Y* → 2^*D*^ such that

(31) $$\begin{array}{c} H^{-1}\left(u\right)=\{\begin{array}{cc} \left(-1,-u-2\right)⋃\left(3,+\infty \right), & \text{if}\;\;-4\leq u\leq -2,\\ \left(-1,u+2\right)⋃\left(3,+\infty \right), & \text{if}\;\;-2<u\leq 0, \end{array} \end{array}$$

which is open in *Y*. Therefore, *H* ^−1^ is compactly open-valued, and hence, (ii) of Theorem 6 is satisfied. Furthermore, define a set-valued mapping *P* : *X* → 2^*Y*^ such that

(32) P−1(y) ={[1,3],if  y=1,[−y+2,1]⋃[3,y+2],if  1<y<2,[0,1]⋃[3,4],if  2≤y≤3,∅,if  y∈(−1,1)⋃(3,+∞).

Now, let *T* : *X* → 2^*Y*^ be defined by

(33) $$\begin{array}{c} T\left(x\right)=\{\begin{array}{cc} \left[2,3\right], & \text{if}\;\;0\leq x<1,\\ \left[1,3\right], & \text{if}\;\;x=1,\\ \left\{1\right\}, & \text{if}\;\;1<x<3,\\ \left[1,3\right], & \text{if}\;\;x=3,\\ \left[2,3\right], & \text{if}\;\;3<x\leq 4. \end{array} \end{array}$$

Then we have $\bar{T(X)}=[1,3]$, which is compact subset of *Y*. Let $K=\bar{T(X)}$. Then (iv_1_) of Theorem 6 is satisfied automatically. In order to check (iii) of Theorem 6, for every *N* = {*u*_0_, *u*_1_,…, *u*_*n*_}∈〈*D*〉, we define a set-valued mapping *φ*_*N*_ : Δ_*n*_ → 2^*X*^ by *φ*_*N*_(*z*) = {0,4} for all *z* ∈ Δ_*n*_. Then (*X*, *D*; *φ*_*N*_) forms an *FWC*-space. For each *y* ∈ (−1, +*∞*), we have

(34) X∖P−1(y)={[0,1)⋃(3,4],if  y=1,[0,−y+2)⋃(y+2,4]⋃(1,3),if  1<y<2,(1,3),if  2≤y≤3[0,4],if  y∈(−1,1)⋃(3,+∞).

We can see that for each *y* ∈ *Y*, *X*∖*P* ^−1^(*y*) is an *FWC*-subspace of (*X*, *D*; *φ*_*N*_) relative to *H*(*y*). Therefore, by Remark 7, we know that (iii) of Theorem 6 holds. Finally, we show that, for each *x* ∈ *X*, *T*(*x*)⊆*P*(*x*) and $T\in \tilde{ℬ}(X,D,Y)$. By the definition of *T*, we can obtain *T* ^−1^ : *Y* → 2^*X*^ as follows:

(35) T−1(y)={0≤x≤4:y∈T(x)}={∅,if  −1<y<1,[1,3],if  y=1,{1,3},if  1<y<2,[3,4]⋃[0,1],if  2≤y≤3,∅,if  3<y<+∞.

Thus, we can easily see that *T* ^−1^(*y*)⊆*P* ^−1^(*y*) for each *y* ∈ *Y*. Hence, for each *x* ∈ *X*, *T*(*x*)⊆*P*(*x*). By using the same method as in Example 4, we can prove that $T\in \tilde{ℬ}(X,D,Y)$. Therefore, all the hypotheses of Theorem 6 are satisfied. We can see that there exists a point $\hat{y}=5/2\in \bar{T(X)}{⋂}_{}K$ such that $H(\hat{y})=\emptyset$.

If *T* = *P* in Theorem 6, then Theorem 6 deduces the following result.

**Corollary 9**

Let (*X*, *D*; *φ*_*N*_) be an FWC-space, *Y* a Hausdorff topological space, and *K* a nonempty compact subset of *Y*. Let *H* : *Y* → 2^*D*^, *Q* : *D* → 2^*X*^, and $T\in \tilde{ℬ}(X,D,Y)$ be set-valued mappings such that

(i) for each *u* ∈ *D*, *H* ^−1^(*u*) is compactly open;
(ii) for each *N* = {*u*_0_, *u*_1_,…, *u*_*n*_}∈〈*D*〉 and each {*u*_*i*_0__, *u*_*i*_1__,…, *u*_*i*_*k*__}⊆*N*,
  (36) $$\begin{array}{c} T\left(\varphi_{N}\left(\Delta_{k}\right)\right)⋂\left(\overset{k}{\underset{j=0}{⋂}}H^{-1}\left(u_{i_{j}}\right)\right)=\emptyset ; \end{array}$$
(iii) one of the following conditions holds:
  (iii_1_) there exists *N*_0_ ∈ 〈*D*〉 such that $\bar{T(X)}\setminus K\subseteq {⋃}_{u\in N_{0}}H^{-1}(u)$ and for each *N* = {*u*_0_, *u*_1_,…, *u*_*n*_}∈〈*D*〉, $\bar{T(\varphi_{N}(\Delta_{n}))}$ is a compact subset of *Y*;
  (iii_2_) for each *N* ∈ 〈*D*〉, there exists a subset *L*_*N*_ of *D* containing *N* such that *Q*(*L*_*N*_) is an *FWC*-subspace of (*X*, *D*; *φ*_*N*_) relative to *L*_*N*_, $\bar{\left(T\circ Q)(L_{N}\right)}$ is a compact subset of *Y*, and
    (37) $$\begin{array}{c} \bar{\left(T\circ Q)(L_{N}\right)}\setminus K\subseteq \underset{u\in L_{N}}{⋃}H^{-1}\left(u\right). \end{array}$$

Then there exists $\hat{y}\in \bar{T(X)}{⋂}_{}K$ such that $H(\hat{y})=\emptyset$.

Taking *X* = *D* and *Q*(*x*) = {*x*} in Corollary 9, we can obtain the following result.

**Corollary 10**

Let (*X*; *φ*_*N*_) be an FWC-space, *Y* a Hausdorff topological space, and *K* a nonempty compact subset of *Y*. Let *H* : *Y* → 2^*X*^ and $T\in \tilde{ℬ}(X,Y)$ be set-valued mappings such that

(i) for each *x* ∈ *X*, *H* ^−1^(*x*) is compactly open;
(ii) for each *N* = {*x*_0_, *x*_1_,…, *x*_*n*_}∈〈*X*〉 and each {*x*_*i*_0__, *x*_*i*_1__,…, *x*_*i*_*k*__}⊆*N*,
  (38) $$\begin{array}{c} T\left(\varphi_{N}\left(\Delta_{k}\right)\right)⋂\left(\overset{k}{\underset{j=0}{⋂}}H^{-1}\left(x_{i_{j}}\right)\right)=\emptyset ; \end{array}$$
(iii) one of the following conditions holds:
  (iii_1_) there exists *N*_0_ ∈ 〈*X*〉 such that $\bar{T(X)}\setminus K\subseteq {⋃}_{x\in N_{0}}H^{-1}(x)$ and for each *N* = {*x*_0_, *x*_1_,…, *x*_*n*_}∈〈*X*〉, $\bar{T(\varphi_{N}(\Delta_{n}))}$ is a compact subset of *Y*;
  (iii_2_) for each *N* ∈ 〈*X*〉, there exists a subset *L*_*N*_ of *X* containing *N* such that *L*_*N*_ is an *FWC*-subspace of (*X*; *φ*_*N*_), $\bar{T(L_{N})}$ is a compact subset of *Y*, and
    (39) $$\begin{array}{c} \bar{T(L_{N})}\setminus K\subseteq \underset{x\in L_{N}}{⋃}H^{-1}\left(x\right). \end{array}$$

Then there exists $\hat{y}\in \bar{T(X)}{⋂}_{}K$ such that $H(\hat{y})=\emptyset$.

## 3. Existence of Solutions to Variational Relation Problem

In 2008, Luc [29] introduced a variational relation problem which unifies many equilibrium problems, optimization problems, and variational or differential inclusion problems. Since then, further studies on variational relation problems were investigated by many authors; see, for example, [30–32] and the references therein.

Let (*X*; *φ*_*N*_) be an *FWC*-space, *Y* a Hausdorff topological space, *K* a nonempty compact subset of *Y*, and $T\in \tilde{ℬ}(X,Y)$ a set-valued mapping. In this section, we will study the following variational relation problems in *FWC*-spaces.

1. Let *R* be a relation linking *y* ∈ *Y* and *x* ∈ *X*. Find $\hat{y}\in \bar{T(X)}{⋂}_{}K$ such that $R(\hat{y},x)$ holds for each *x* ∈ *X*.
2. Let *Z* be a nonempty set, *Q* : *X* → 2^*Z*^ a set-valued mapping, and *R* a relation linking *y* ∈ *Y* and *z* ∈ *Z*. Find $\hat{y}\in \bar{T(X)}{⋂}_{}K$ such that $R(\hat{y},z)$ holds for each *x* ∈ *X* and each *z* ∈ *Q*(*x*).

By applying Corollary 10, we have the following existence theorem of solutions to the variational relation problem in *FWC*-spaces.

**Theorem 11**

Let (*X*; *φ*_*N*_) be an FWC-space, *Y* a Hausdorff topological space, and *K* a nonempty compact subset of *Y*. Let $T\in \tilde{ℬ}(X,Y)$ be a set-valued mapping and let *R* be a relation linking elements *x* ∈ *X*, *y* ∈ *Y* such that

(i) for each *x* ∈ *X*, the set {*y* ∈ *Y* : *R*(*y*, *x*)  *does* *not* *hold*} is compactly open;
(ii) for each *N* = {*x*_0_, *x*_1_,…, *x*_*n*_}∈〈*X*〉, each {*x*_*i*_0__, *x*_*i*_1__,…, *x*_*i*_*k*__}⊆*N* and each *y* ∈ *T*(*φ*_*N*_(Δ_*k*_)), there exists $\bar{j}\in \{0,1,\ldots ,k\}$ such that $R(y,x_{i_{\bar{j}}})$ holds;
(iii) one of the following conditions holds:
  (iii_1_) there exists *N*_0_ ∈ 〈*X*〉 such that $\bar{T(X)}\setminus K\subseteq {⋃}_{x\in N_{0}}\{y\in Y:R(y,x)\;\;does\;\;not\;\;hold\}$ and for each *N* = {*x*_0_, *x*_1_,…, *x*_*n*_}∈〈*X*〉, $\bar{T(\varphi_{N}(\Delta_{n}))}$ is a compact subset of *Y*;
  (iii_2_) for each *N* ∈ 〈*X*〉, there exists a subset *L*_*N*_ of *X* containing *N* such that *L*_*N*_ is an *FWC*-subspace of (*X*; *φ*_*N*_), $\bar{T(L_{N})}$ is a compact subset of *Y*, and
    (40) $$\begin{array}{c} \bar{T(L_{N})}\setminus K\subseteq \underset{x\in L_{N}}{⋃}\left\{y\in Y:R\left(y,x\right)\;\;does\;\;not\;\;hold\right\}. \end{array}$$

Then there exists $\hat{y}\in \bar{T(X)}{⋂}_{}K$ such that $R(\hat{y},x)$ holds for each *x* ∈ *X*.

**Proof**

Define a set-valued mapping *H* : *Y* → 2^*X*^ by

(41) $$\begin{array}{c} H\left(y\right)=\left\{x\in X:R\left(y,x\right)\;\;\text{does}\;\;\text{not}\;\;\text{hold}\right\}\;\text{for}\;\;\text{each}\;\;y\in Y. \end{array}$$

By (i), for each *x* ∈ *X*, *H* ^−1^(*x*) is compactly open. By (ii), for each *N* = {*x*_0_, *x*_1_,…, *x*_*n*_}∈〈*X*〉 and each {*x*_*i*_0__, *x*_*i*_1__,…, *x*_*i*_*k*__}⊆*N*, we have

(42) $$\begin{array}{c} T\left(\varphi_{N}\left(\Delta_{k}\right)\right)\subseteq \overset{k}{\underset{j=0}{⋃}}\left(Y\setminus H^{-1}\left(x_{i_{j}}\right)\right), \end{array}$$

which implies that

(43) $$\begin{array}{c} T\left(\varphi_{N}\left(\Delta_{k}\right)\right)⋂\left(\overset{k}{\underset{j=0}{⋂}}H^{-1}\left(x_{i_{j}}\right)\right)=\emptyset . \end{array}$$

By (iii), we know that one of the following conditions holds:

(a) there exists *N*_0_ ∈ 〈*X*〉 such that $\bar{T(X)}\setminus K\subseteq {⋃}_{x\in N_{0}}H^{-1}(x)$ and for each *N* = {*x*_0_, *x*_1_,…, *x*_*n*_}∈〈*X*〉, $\bar{T(\varphi_{N}(\Delta_{n}))}$ is a compact subset of *Y*;
(b) for each *N* ∈ 〈*X*〉, there exists a subset *L*_*N*_ of *X* containing *N* such that *L*_*N*_ is an *FWC*-subspace of (*X*; *φ*_*N*_), $\bar{T(L_{N})}$ is a compact subset of *Y*, and
  (44) $$\begin{array}{c} \bar{T(L_{N})}\setminus K\subseteq \underset{x\in L_{N}}{⋃}H^{-1}\left(x\right). \end{array}$$

Therefore, by Corollary 10, there exists $\hat{y}\in \bar{T(X)}{⋂}_{}K$ such that $H(\hat{y})=\emptyset$; that is, $R(\hat{y},x)$ holds for each *x* ∈ *X*.

By taking *X* = *Y* and *T*(*x*) = {*x*} for every *x* ∈ *X* in Theorem 11, we can obtain the following result.

**Corollary 12**

Let (*X*; *φ*_*N*_) be an FWC-space and *K* a nonempty compact subset of *X*, where *X* is a Hausdorff topological space. Let $I\in \tilde{ℬ}(X,X)$, where *I* is the identity mapping on *X*. Let *R* be a relation linking elements *x* ∈ *X*, *y* ∈ *X* such that

(i) for each *x* ∈ *X*, the set {*y* ∈ *X* : *R*(*y*, *x*)  *does* *not* *hold*} is compactly open;
(ii) for each *N* = {*x*_0_, *x*_1_,…, *x*_*n*_}∈〈*X*〉, each {*x*_*i*_0__, *x*_*i*_1__,…, *x*_*i*_*k*__}⊆*N* and each *y* ∈ *φ*_*N*_(Δ_*k*_), there exists $\bar{j}\in \{0,1,\ldots ,k\}$ such that $R(y,x_{i_{\bar{j}}})$ holds;
(iii) one of the following conditions holds:
  (iii_1_) there exists *N*_0_ ∈ 〈*X*〉 such that *X*∖*K*⊆⋃_*x*∈*N*_0__{*y* ∈ *X* : *R*(*y*, *x*)  *does* *not* *hold*} and *φ*_*N*_ : Δ_*n*_ → 2^*X*^ is a compact set-valued mapping for each *N* = {*u*_0_, *u*_1_,…, *u*_*n*_}∈〈*D*〉;
  (iii_2_) for each *N* ∈ 〈*X*〉, there exists a compact subset *L*_*N*_ of *X* containing *N* such that *L*_*N*_ is an *FWC*-subspace of (*X*; *φ*_*N*_) and
    (45) $$\begin{array}{c} L_{N}\setminus K\subseteq \underset{x\in L_{N}}{⋃}\left\{y\in X:R\left(y,x\right)\;\;does\;\;not\;\;hold\right\}. \end{array}$$

Then there exists $\hat{y}\in K$ such that $R(\hat{y},x)$ holds for each *x* ∈ *X*.

**Corollary 13**

Let (*X*; *φ*_*N*_) be an FWC-space, where *X* is a Hausdorff compact topological space. Let $I\in \tilde{ℬ}(X,X)$, where *I* is the identity mapping on *X*. Let *R* be a relation linking elements *x* ∈ *X*, *y* ∈ *X* such that the following conditions hold:

- for each *x* ∈ *X*, the set {*y* ∈ *X* : *R*(*y*, *x*)  *does* *not* *hold*} is compactly open;
- for each *N* = {*x*_0_, *x*_1_,…, *x*_*n*_}∈〈*X*〉, each {*x*_*i*_0__, *x*_*i*_1__,…, *x*_*i*_*k*__}⊆*N* and each *y* ∈ *φ*_*N*_(Δ_*k*_), there exists $\bar{j}\in \{0,1,\ldots ,k\}$ such that $R(y,x_{i_{\bar{j}}})$ holds.

Then there exists $\hat{y}\in X$ such that $R(\hat{y},x)$ holds for each *x* ∈ *X*.

**Proof**

Let *K* = *X*. Then (iii_1_) of Corollary 12 is satisfied automatically. Hence, the conclusion of Corollary 13 follows from Corollary 12.

**Remark 14**

It is interesting to compare Corollary 13 with Theorem 2.1 of Pu and Yang [32] in the following aspects: (1) (i) of Corollary 13 is weaker than (i) of Theorem 2.1 of Pu and Yang [32], which can be stated as follows: for each *x* ∈ *X*, the set {*y* ∈ *X* : *R*(*y*, *x*)  holds} is closed; (2) (ii) of Theorem 2.1 of Pu and Yang [32] can be stated as follows: for each {*x*_0_, *x*_1_,…, *x*_*n*_}∈〈*X*〉, there exists a continuous mapping *φ*_*N*_ : Δ_*n*_ → *X* such that, for each *λ* = {*λ*_0_, *λ*_1_,…, *λ*_*n*_} ∈ Δ_*n*_, there exists *i* ∈ *J*(*λ*) such that *R*(*φ*_*N*_(*λ*), *x*_*i*_) holds, where *J*(*λ*) = {*i* ∈ {0,1,…, *n*} : *λ*_*i*_ > 0}. By (ii) of Theorem 2.1 of Pu and Yang [32], we know that (*X*, *φ*_*N*_) in Theorem 2.1 of Pu and Yang [32] forms an *FWC*-space; (3) in Corollary 13, the topological space *X* needs not to have the fixed point property, but *X* in Theorem 2.1 of Pu and Yang [32] needs to possess the fixed point property; (4) for the identity mapping *I* on *X* in Theorem 2.1 of Pu and Yang [32], we must have $I\in \tilde{ℬ}(X,X)$. In fact, for every *N* = {*x*_0_, *x*_1_,…, *x*_*n*_}∈〈*X*〉 and for every continuous mapping *ψ* : *φ*_*N*_(Δ_*n*_) → Δ_*n*_, the composition *ψ*∘*φ*_*N*_ : Δ_*n*_ → Δ_*n*_ is continuous, where *φ*_*N*_ coincides with the one in (ii) of Theorem 2.1 of Pu and Yang [32]. Then by Brouwer fixed point theorem, there exists *z*_0_ ∈ Δ_*n*_ such that *z*_0_ = *ψ*∘*φ*_*N*_(*z*_0_), which implies that $I\in \tilde{ℬ}(X,X)$.

**Theorem 15**

Let (*X*; *φ*_*N*_) be an FWC-space, *Y* a Hausdorff topological space, *K* a nonempty compact subset of *Y*, and *Z* a nonempty set. Let $T\in \tilde{ℬ}(X,Y)$, *Q* : *X* → 2^*Z*^ be set-valued mappings and *R* a relation linking elements *y* ∈ *Y*, *z* ∈ *Z*. Assume that

(i) for each *x* ∈ *X*, the set {*y* ∈ *Y* : *P*(*y*)⋂*Q*(*x*) ≠ *∅*} is compactly open, where *P* : *Y* → 2^*Z*^ is defined by *P*(*y*) = {*z* ∈ *Z* : *R*(*y*, *z*)  *does* *not* *hold*} for each *y* ∈ *Y*;
(ii) for each *N* = {*x*_0_, *x*_1_,…, *x*_*n*_}∈〈*X*〉, each {*x*_*i*_0__, *x*_*i*_1__,…, *x*_*i*_*k*__}⊆*N* and each *y* ∈ *T*(*φ*_*N*_(Δ_*k*_)), there exists $\bar{j}\in \{0,1,\ldots ,k\}$ such that *R*(*y*, *z*) holds for each $z\in Q(x_{i_{\bar{j}}})$;
(iii) one of the following conditions holds:
  (iii_1_) there exists *N*_0_ ∈ 〈*X*〉 such that $\bar{T(X)}\setminus K\subseteq {⋃}_{x\in N_{0}}\{y\in Y:P(y){⋂}_{}Q(x)\neq \emptyset \}$ and for each *N* = {*x*_0_, *x*_1_,…, *x*_*n*_}∈〈*X*〉, $\bar{T(\varphi_{N}(\Delta_{n}))}$ is a compact subset of *Y*;
  (iii_2_) for each *N* ∈ 〈*X*〉, there exists a subset *L*_*N*_ of *X* containing *N* such that *L*_*N*_ is an *FWC*-subspace of (*X*; *φ*_*N*_), $\bar{T(L_{N})}$ is a compact subset of *Y*, and
    (46) $$\begin{array}{c} \bar{T(L_{N})}\setminus K\subseteq \underset{x\in L_{N}}{⋃}\left\{y\in Y:P\left(y\right)⋂Q\left(x\right)\neq \emptyset \right\}. \end{array}$$

Then there exists $\hat{y}\in \bar{T(X)}{⋂}_{}K$ such that $R(\hat{y},z)$ holds for each *x* ∈ *X* and each *z* ∈ *Q*(*x*).

**Proof**

Let the relation $\tilde{R}$ on *Y* and *X* be defined by $\tilde{R}(y,x)$ holds if and only if *P*(*y*)⋂*Q*(*x*) = *∅*. Then by (i), for each *x* ∈ *X*, the set $\left\{y\in Y:\tilde{R}(y,x)\;\;\text{does}\;\;\text{not}\;\;\text{hold}\right\}$ is compactly open. By (ii), for each *N* = {*x*_0_, *x*_1_,…, *x*_*n*_}∈〈*X*〉, each {*x*_*i*_0__, *x*_*i*_1__,…, *x*_*i*_*k*__}⊆*N* and each *y* ∈ *T*(*φ*_*N*_(Δ_*k*_)), there exists $\bar{j}\in \{0,1,\ldots ,k\}$ such that

(47) Q(xij¯)⊆{z∈Z:R(y,z)  holds}=Z∖{z∈Z:R(y,z)  does  not  hold}=Z∖P(y);

that is, $Q(x_{i_{\bar{j}}}){⋂}_{}P(y)=\emptyset$. Thus, $\tilde{R}(y,x_{i_{\bar{j}}})$ holds. By (iii), we know that one of the following conditions holds:

(a) there exists *N*_0_ ∈ 〈*X*〉 such that $\bar{T(X)}\setminus K\subseteq {⋃}_{x\in N_{0}}\{y\in Y:\tilde{R}(y,x)\;\;\text{does}\;\;\text{not}\;\;\text{hold}\}$ and for each *N* = {*x*_0_, *x*_1_,…, *x*_*n*_}∈〈*X*〉, $\bar{T(\varphi_{N}(\Delta_{n}))}$ is compact subset of *Y*;
(b) for each *N* ∈ 〈*X*〉, there exists a subset *L*_*N*_ of *X* containing *N* such that *L*_*N*_ is an *FWC*-subspace of (*X*; *φ*_*N*_) and
  (48) $$\begin{array}{c} \bar{T(L_{N})}\setminus K\subseteq \underset{x\in L_{N}}{⋃}\left\{y\in Y:\tilde{R}\left(y,x\right)\;\;\text{does}\;\;\text{not}\;\;\text{hold}\right\}, \end{array}$$

where $\bar{T(L_{N})}$ is a compact subset of *Y*. Therefore, by Theorem 11, there exists $\hat{y}\in \bar{T(X)}{⋂}_{}K$ such that $\tilde{R}(\hat{y},x)$ holds for each *x* ∈ *X*; that is, $R(\hat{y},z)$ holds for each *x* ∈ *X* and each *z* ∈ *Q*(*x*).

**Remark 16**

(1)Theorem 15 generalizes Theorem 3.1 of Balaj and Lin [30] in the following aspects: (a) The underlying spaces of Theorem 15 and Theorem 3.1 of Balaj and Lin [30] are *FWC*-spaces and convex spaces, respectively. It follows from the previous analysis that *FWC*-spaces include convex spaces as special cases; (b) The class of better admissible mappings in Theorem 15 and Theorem 3.1 of Balaj and Lin [30] are $\tilde{ℬ}(X,Y)$ and *ℬ*(*X*, *Y*), respectively. By Remark 3, we know that *ℬ*(*X*, *Y*) is contained in $\tilde{ℬ}(X,Y)$; (c) *Z* in Theorem 15 does not possess any topological structure; (d) *T* in Theorem 15 needs not to be compact. In fact, if *T* in Theorem 15 is compact, then we know that (iii_1_) of Theorem 15 is satisfied by taking $K=\bar{T(X)}$; (e) (ii) of Theorem 3.1 of Balaj and Lin [30] can be stated as follows: for each *N* = {*x*_0_, *x*_1_,…, *x*_*n*_}∈〈*X*〉, each *x* ∈ co⁡{*x*_0_, *x*_1_,…, *x*_*n*_} and each *y* ∈ *T*(*x*), there exists $\bar{j}\in \{0,1,\ldots ,n\}$ such that *R*(*y*, *z*)holds for all *z* ∈ *Q*(*x*_*j*_). (ii) of Theorem 15 is weaker than (ii) of Theorem 3.1 of Balaj and Lin [30]. In fact, for every *N* = {*x*_0_, *x*_1_,…, *x*_*n*_}∈〈*X*〉, we can define a continuous mapping *φ*_*N*_ : Δ_*n*_ → co⁡(*N*)⊆*X* by

(49) φN(∑j=0nλjej)=∑j=0nλjxjfor  each  (λ0,λ1,…,λn)=∑j=0nλjej∈Δn.

Therefore, (*X*; *φ*_*N*_) forms an *FWC*-space. On the basis of this fact, we can see that (ii) of Theorem 3.1 of Balaj and Lin [30] implies (ii) of Theorem 15.

(2) Theorem 15 is equivalent to Theorem 11. In fact, we have shown that Theorem 11 implies Theorem 15. Conversely, if *X* = *Z* and *Q*(*x*) = {*x*} for each *x* ∈ *X* in Theorem 15, then Theorem 15 becomes Theorem 11.

**Corollary 17**

Let *P* : *Y* → 2^*Z*^ be defined by *P*(*y*) = {*z* ∈ *Z* : *R*(*y*, *z*)  *does* *not* *hold*} for each *y* ∈ *Y*. Theorem 15 is true if (i) of Theorem 15 is replaced by one of the following conditions:

(i)′ for each *z* ∈ *Q*(*X*), the set {*y* ∈ *Y* : *R*(*y*, *z*)  *hold* *s*} is compactly closed;
(ii)′ *Z* is a topological space, the set-valued mapping *P* is lower semicontinuous, and *Q* has open values.

**Proof**

Suppose that (i)′ is satisfied. Then ⋂_*z*∈*Q*(*x*)_{*y* ∈ *Y* : *R*(*y*, *z*)  holds} is compactly closed for each *x* ∈ *X*. Thus, {*y* ∈ *Y* : *P*(*y*)⋂*Q*(*x*) ≠ *∅*} = *Y*∖⋃_*z*∈*Q*(*x*)_{*R*(*y*, *z*)  does  not  hold} is compactly open. If (ii)′ holds, then by the definition of a lower semicontinuous set-valued mapping, for each *x* ∈ *X*, the set {*y* ∈ *Y* : *P*(*y*)⋂*Q*(*x*) ≠ *∅*} = *Y*∖{*y* ∈ *Y* : *P*(*y*)⋂*Q*(*x*) = *∅*} is open and thus, compactly open.

## 4. Generalized Equilibrium Theorems

In recent years, many authors (see, e.g., [33–35] and the references therein) studied one or more of the following generalized equilibrium problems.

Let *D* and *Z* be nonempty sets and *Y* a topological space. Let *L* : *Y* × *D* → 2^*Z*^ and *W* : *Y* → 2^*Z*^ be set-valued mappings. Find $\hat{y}\in Y$ such that one of the following situations occurs:

(50) $$\begin{array}{c} L\left(\hat{y},u\right)\subseteq W\left(\hat{y}\right)\;\text{for}\;\;\text{each}\;\;u\in D,\\ L\left(\hat{y},u\right)⊈W\left(\hat{y}\right)\;\text{for}\;\;\text{each}\;\;u\in D,\\ L\left(\hat{y},u\right)⋂W\left(\hat{y}\right)\neq \emptyset \;\text{for}\;\;\text{each}\;\;u\in D,\\ L\left(\hat{y},u\right)⋂W\left(\hat{y}\right)=\emptyset \;\text{for}\;\;\text{each}\;\;u\in D. \end{array}$$

Let *E* be another nonempty set, *G* : *Y* → 2^*E*^ a set-valued mapping, and *ξ* : *E* × *Y* × *D* → *Z* a single-valued mapping. The generalized implicit vector equilibrium problem is to find $\hat{y}\in Y$ such that, for each *u* ∈ *D*, there exists $\bar{s}\in G(\hat{y})$ satisfying $\xi (\bar{s},\hat{y},u)\notin W(\hat{y})$. For more details, the reader may consult [35] and the references therein.

In this section, as applications of Theorem 6, we will prove new existence theorems of solutions to generalized equilibrium problems in *FWC*-spaces without any linear, convex, and topological structure.

**Theorem 18**

Let (*X*, *D*; *φ*_*N*_) be an *FWC*-space, *Y* a Hausdorff topological space, *K* a nonempty compact subset of *Y*, and *Z* a nonempty set. Let *J* : *Y* × *X* → 2^*Z*^, *L* : *Y* × *D* → 2^*Z*^, *F*, *W* : *Y* → 2^*Z*^, *Q* : *D* → 2^*X*^, and $T\in \tilde{ℬ}(X,D,Y)$ be set-valued mappings such that

(i) for each *x* ∈ *X* and each *y* ∈ *T*(*x*), *J*(*y*, *x*)⊆*F*(*y*);
(ii) for each *u* ∈ *D*, the set {*y* ∈ *Y* : *L*(*y*, *u*)⊆*W*(*y*)} is compactly closed;
(iii) for each *y* ∈ *Y*, the set {*x* ∈ *X* : *J*(*y*, *x*)⊈*F*(*y*)} is an *FWC*-subspace of (*X*, *D*; *φ*_*N*_) relative to the set {*u* ∈ *D* : *L*(*y*, *u*)⊈*W*(*y*)};
(iv) one of the following conditions holds:
  (iv_1_) *Y*∖*K*⊆⋃_*u*∈*N*_0__{*y* ∈ *Y* : *L*(*y*, *u*)⊈*W*(*y*)} for some *N*_0_ ∈ 〈*D*〉 and for each *N* = {*u*_0_, *u*_1_,…, *u*_*n*_}∈〈*D*〉, $\bar{T(\varphi_{N}(\Delta_{n}))}$ is a compact subset of *Y*;
  (iv_2_) for each *N* ∈ 〈*D*〉, there exists a subset *L*_*N*_ of *D* containing *N* such that *Q*(*L*_*N*_) is an *FWC*-subspace of (*X*, *D*; *φ*_*N*_) relative to *L*_*N*_, $\bar{\left(T\circ Q)(L_{N}\right)}$ is a compact subset of *Y*, and
    (51) $$\begin{array}{c} \bar{\left(T\circ Q)(L_{N}\right)}\setminus K\subseteq \underset{u\in L_{N}}{⋃}\left\{y\in Y:L\left(y,u\right)⊈W\left(y\right)\right\}. \end{array}$$

Then there exists $\hat{y}\in \bar{T(X)}{⋂}_{}K$ such that $L(\hat{y},u)\subseteq W(\hat{y})$ for each *u* ∈ *D*.

**Proof**

Define *P* : *X* → 2^*Y*^ and *H* : *Y* → 2^*D*^ by

(52) $$\begin{array}{c} P\left(x\right)=\left\{y\in Y:J\left(y,x\right)\subseteq F\left(y\right)\right\}\;\text{for}\;\;\text{each}\;\;x\in X,\\ H\left(y\right)=\left\{u\in D:L\left(y,u\right)⊈W\left(y\right)\right\}\;\text{for}\;\;\text{each}\;\;y\in Y. \end{array}$$

By (i), we have *T*(*x*)⊆*P*(*x*) for each *x* ∈ *X*. By (ii), for each *u* ∈ *D*, *H* ^−1^(*u*) is compactly open. Now, we show that (iii) of Theorem 6 is satisfied. Suppose the contrary. Then there exist *N* = {*u*_0_, *u*_1_,…, *u*_*n*_}∈〈*D*〉 and {*u*_*i*_0__, *u*_*i*_1__,…, *u*_*i*_*k*__}⊆*N* such that

(53) $$\begin{array}{c} P\left(\varphi_{N}\left(\Delta_{k}\right)\right)\cap \left(\overset{k}{\underset{j=0}{⋂}}H^{-1}\left(u_{i_{j}}\right)\right)\neq \emptyset , \end{array}$$

which implies that there exists y* ∈ *P*(*φ*_*N*_(Δ_*k*_)) such that y* ∈ *H* ^−1^(*u*_*i*_*j*__) for each *j* ∈ {0,1,…, *k*}; that is, *u*_*i*_*j*__ ∈ {*u* ∈ *D* : *L*(y*, *u*)⊈  *W*(y*)}. By (iii), we have

(54) $$\begin{array}{c} \varphi_{N}\left(\Delta_{k}\right)\subseteq \left\{x\in X:J\left(y^{*},x\right)⊈F\left(y^{*}\right)\right\}, \end{array}$$

which implies that

(55) $$\begin{array}{c} J\left(y^{*},x\right)⊈F\left(y^{*}\right)\;\text{for}\;\;\text{each}\;\;x\in \varphi_{N}\left(\Delta_{k}\right). \end{array}$$

Since y* ∈ *P*(*φ*_*N*_(Δ_*k*_)), it follows that there exists $\bar{x}\in \varphi_{N}(\Delta_{k})$ such that $y^{*}\in P(\bar{x})$; that is, $J(y^{*},\bar{x})\subseteq F(y^{*})$, which contradicts (55). Therefore, (iii) of Theorem 6 holds. Suppose that (iv_1_) of Theorem 18 is fulfilled. Then by (iv_1_) and the definition of *H*, we know that there exists *N*_0_ ∈ 〈*D*〉 such that *Y*∖*K*⊆⋃_*d*∈*N*_0__ *H* ^−1^(*d*) and for each *N* = {*u*_0_, *u*_1_,…, *u*_*n*_}∈〈*D*〉, $\bar{T(\varphi_{N}(\Delta_{n}))}$ is a compact subset of *Y*. Therefore, (iv_1_) of Theorem 6 is satisfied. If (iv_2_) of Theorem 18 holds, then by (iv_2_) and the definition of *H* again, we know that for each *N* ∈ 〈*D*〉, there exists a subset *L*_*N*_ of *D* containing *N* such that *Q*(*L*_*N*_) is an *FWC*-subspace of (*X*, *D*; *φ*_*N*_) relative to *L*_*N*_ and

(56) $$\begin{array}{c} \bar{\left(T\circ Q)(L_{N}\right)}\setminus K\subseteq \underset{u\in L_{N}}{⋃}H^{-1}\left(u\right), \end{array}$$

where $\bar{\left(T\circ Q)(L_{N}\right)}$ is a compact subset of *Y*. Therefore, (iv_2_) of Theorem 6 is satisfied. Thus, all conditions of Theorem 6 are fulfilled. By Theorem 6, there exists a point $\hat{y}\in \bar{T(X)}{⋂}_{}K$ such that $H(\hat{y})=\emptyset$; that is, there exists a point $\hat{y}\in \bar{T(X)}{⋂}_{}K$ such that $L(\hat{y},u)\subseteq W(\hat{y})$ for each *u* ∈ *D*. This completes the proof.

**Remark 19**

Theorem 18 generalizes Theorem 4.1 of Fang and Huang [34] in the following aspects: (a) The underlying spaces of Theorem 18 and Theorem 4.1 of Fang and Huang [34] are *FWC*-spaces and *FC*-spaces, respectively. By the previous analysis, we know that *FWC*-spaces include *FC*-spaces as special cases; (b) The class of better admissible mappings in Theorem 18 and Theorem 4.1 of Fang and Huang [34] are $\tilde{ℬ}(X,D,Y)$ and *ℬ*(*X*, *Y*), respectively. By Remark 3, we know that *ℬ*(*X*, *Y*) is contained in $\tilde{ℬ}(X,D,Y)$; (c) (ii) of Theorem 18 is weaker than (i) of Theorem 4.1 of Fang and Huang [34]; (d) (iii) of Theorem 18 is weaker than (iii) of Theorem 4.1 of Fang and Huang [34]; (e) (iv_2_) of Theorem 18 is weaker than (iv) of Theorem 4.1 of Fang and Huang [34]. It should be emphasized that the proof of Theorem 18 is different from that of Theorem 4.1 of Fang and Huang [34].

By using the same argument as in Theorem 18, we can obtain Theorems 20, 22, and 23. We omit their proofs.

**Theorem 20**

Let (*X*, *D*; *φ*_*N*_) be an *FWC*-space, *Y* a Hausdorff topological space, *K* a nonempty compact subset of *Y*, and *Z* a nonempty set. Let *J* : *Y* × *X* → 2^*Z*^, *L* : *Y* × *D* → 2^*Z*^, *F*, *W* : *Y* → 2^*Z*^, *Q* : *D* → 2^*X*^, and $T\in \tilde{ℬ}(X,D,Y)$ be set-valued mappings. Assume that

(i) for each *x* ∈ *X* and each *y* ∈ *T*(*x*), *J*(*y*, *x*)⊈*F*(*y*);
(ii) for each *u* ∈ *D*, the set {*y* ∈ *Y* : *L*(*y*, *u*)⊈*W*(*y*)} is compactly closed;
(iii) for each *y* ∈ *Y*, the set {*x* ∈ *X* : *J*(*y*, *x*)⊆*F*(*y*)} is an *FWC*-subspace of (*X*, *D*; *φ*_*N*_) relative to the set {*u* ∈ *D* : *L*(*y*, *u*)⊆*W*(*y*)};
(iv) one of the following conditions holds:
  (iv_1_) *Y*∖*K*⊆⋃_*u*∈*N*_0__{*y* ∈ *Y* : *L*(*y*, *u*)⊆*W*(*y*)} for some *N*_0_ ∈ 〈*D*〉 and for each *N* = {*u*_0_, *u*_1_,…, *u*_*n*_}∈〈*D*〉, $\bar{T(\varphi_{N}(\Delta_{n}))}$ is a compact subset of *Y*;
  (iv_2_) for each *N* ∈ 〈*D*〉, there exists a subset *L*_*N*_ of *D* containing *N* such that *Q*(*L*_*N*_) is an *FWC*-subspace of (*X*, *D*; *φ*_*N*_) relative to *L*_*N*_, $\bar{\left(T\circ Q)(L_{N}\right)}$ is a compact subset of *Y*, and
    (57) $$\begin{array}{c} \bar{\left(T\circ Q)(L_{N}\right)}\setminus K\subseteq \underset{u\in L_{N}}{⋃}\left\{y\in Y:L\left(y,u\right)\subseteq W\left(y\right)\right\}. \end{array}$$

Then there exists $\hat{y}\in \bar{T(X)}{⋂}_{}K$ such that $L(\hat{y},u)⊈W(\hat{y})$ for each *u* ∈ *D*.

**Remark 21**

Theorem 20 generalizes Theorem 4.3 of Fang and Huang [34] in the following aspects: (a) The underlying spaces of Theorem 18 and Theorem 4.3 of Fang and Huang [34] are *FWC*-spaces and *FC*-spaces, respectively. It follows from the previous analysis that *FWC*-spaces include *FC*-spaces as special cases; (b) The class of better admissible mappings in Theorem 18 and Theorem 4.3 of Fang and Huang [34] are $\tilde{ℬ}(X,D,Y)$ and *ℬ*(*X*, *Y*), respectively. It follows from Remark 3 that *ℬ*(*X*, *Y*) is contained in $\tilde{ℬ}(X,D,Y)$; (c) (ii) of Theorem 18 is weaker than (i) and (ii) of Theorem 4.3 of Fang and Huang [34]; (d) (iii) of Theorem 18 is weaker than (v) of Theorem 4.3 of Fang and Huang [34]; (e) (iv_2_) of Theorem 18 is weaker than (vi) of Theorem 4.3 of Fang and Huang [34].

**Theorem 22**

Let (*X*, *D*; *φ*_*N*_) be an *FWC*-space, *Y* a Hausdorff topological space, *K* a nonempty compact subset of *Y*, and *Z* a nonempty set. Let *J* : *Y* × *X* → 2^*Z*^, *L* : *Y* × *D* → 2^*Z*^, *F*, *W* : *Y* → 2^*Z*^, *Q* : *D* → 2^*X*^, and $T\in \tilde{ℬ}(X,D,Y)$ be set-valued mappings. Assume that

(i) for each *x* ∈ *X* and each *y* ∈ *T*(*x*), *J*(*y*, *x*)⋂*F*(*y*) ≠ *∅*;
(ii) for each *u* ∈ *D*, the set {*y* ∈ *Y* : *L*(*y*, *u*)⋂*W*(*y*) ≠ *∅*} is compactly closed;
(iii) for each *y* ∈ *Y*, the set {*x* ∈ *X* : *J*(*y*, *x*)⋂*F*(*y*) = *∅*} is an *FWC*-subspace of (*X*, *D*; *φ*_*N*_) relative to the set {*u* ∈ *D* : *L*(*y*, *u*)⋂*W*(*y*) = *∅*};
(iv) one of the following conditions holds:
  (iv_1_) *Y*∖*K*⊆⋃_*u*∈*N*_0__{*y* ∈ *Y* : *L*(*y*, *u*)⋂*W*(*y*) = *∅*} for some *N*_0_ ∈ 〈*D*〉 and for each *N* = {*u*_0_, *u*_1_,…, *u*_*n*_}∈〈*D*〉, $\bar{T(\varphi_{N}(\Delta_{n}))}$ is a compact subset of *Y*;
  (iv_2_) for each *N* ∈ 〈*D*〉, there exists a subset *L*_*N*_ of *D* containing *N* such that *Q*(*L*_*N*_) is an *FWC*-subspace of (*X*, *D*; *φ*_*N*_) relative to *L*_*N*_, $\bar{\left(T\circ Q)(L_{N}\right)}$ is a compact subset of *Y*, and
    (58) $$\begin{array}{c} \bar{\left(T\circ Q)(L_{N}\right)}\setminus K\subseteq \underset{u\in L_{N}}{⋃}\left\{y\in Y:L\left(y,u\right)⋂W\left(y\right)=\emptyset \right\}. \end{array}$$

Then there exists $\hat{y}\in \bar{T(X)}{⋂}_{}K$ such that $L(\hat{y},u){⋂}_{}W(\hat{y})\neq \emptyset$ for each *u* ∈ *D*.

**Theorem 23**

Let (*X*, *D*; *φ*_*N*_) be an *FWC*-space, *Y* a Hausdorff topological space, *K* a nonempty compact subset of *Y*, and *Z* a nonempty set. Let *J* : *Y* × *X* → 2^*Z*^, *L* : *Y* × *D* → 2^*Z*^, *F*, *W* : *Y* → 2^*Z*^, *Q* : *D* → 2^*X*^, and $T\in \tilde{ℬ}(X,D,Y)$ be set-valued mappings. Assume that

(i) for each *x* ∈ *X* and each *y* ∈ *T*(*x*), *J*(*y*, *x*)⋂*F*(*y*) = *∅*;
(ii) for each *u* ∈ *D*, the set {*y* ∈ *Y* : *L*(*y*, *u*)⋂*W*(*y*) = *∅*} is compactly closed;
(iii) for each *y* ∈ *Y*, the set {*x* ∈ *X* : *J*(*y*, *x*)⋂*F*(*y*) ≠ *∅*} is an *FWC*-subspace of (*X*, *D*; *φ*_*N*_) relative to the set {*u* ∈ *D* : *L*(*y*, *u*)⋂*W*(*y*) ≠ *∅*};
(iv) one of the following conditions holds:
  (iv_1_) *Y*∖*K*⊆⋃_*u*∈*N*_0__{*y* ∈ *Y* : *L*(*y*, *u*)⋂*W*(*y*) ≠ *∅*} for some *N*_0_ ∈ 〈*D*〉 and for each *N* = {*u*_0_, *u*_1_,…, *u*_*n*_}∈〈*D*〉, $\bar{T(\varphi_{N}(\Delta_{n}))}$ is a compact subset of *Y*;
  (iv_2_) for each *N* ∈ 〈*D*〉, there exists a subset *L*_*N*_ of *D* containing *N* such that *Q*(*L*_*N*_) is an *FWC*-subspace of (*X*, *D*; *φ*_*N*_) relative to *L*_*N*_, $\bar{\left(T\circ Q)(L_{N}\right)}$ is a compact subset of *Y*, and
    (59) $$\begin{array}{c} \bar{\left(T\circ Q)(L_{N}\right)}\setminus K\subseteq \underset{u\in L_{N}}{⋃}\left\{y\in Y:L\left(y,u\right)⋂W\left(y\right)\neq \emptyset \right\}. \end{array}$$

Then there exists $\hat{y}\in \bar{T(X)}{⋂}_{}K$ such that $L(\hat{y},u){⋂}_{}W(\hat{y})=\emptyset$ for each *u* ∈ *D*.

By Theorem 20, we can obtain the following existence theorem of solutions to the generalized implicit vector equilibrium problem.

**Theorem 24**

Let (*X*, *D*; *φ*_*N*_) be an *FWC*-space, *Y* a Hausdorff topological space, *K* a nonempty compact subset of *Y*, and *Z* a nonempty set. Let *F*, *W* : *Y* → 2^*Z*^, *Q* : *D* → 2^*X*^, and $T\in \tilde{ℬ}(X,D,Y)$ be set-valued mappings. Let *E* be a nonempty set and *G* : *Y* → 2^*E*^ a set-valued mapping. Let *ζ* : *E* × *Y* × *X* → *Z* and *ξ* : *E* × *Y* × *D* → *Z* be two single-valued mappings. Assume that

(i) for each *x* ∈ *X* and each *y* ∈ *T*(*x*), *ζ*(*G*(*y*), *y*, *x*)⊈*F*(*y*);
(ii) for each *u* ∈ *D*, the set {*y* ∈ *Y* : *ξ*(*G*(*y*), *y*, *u*)⊈*W*(*y*)} is compactly closed;
(iii) for each *y* ∈ *Y*, the set {*x* ∈ *X* : *ζ*(*G*(*y*), *y*, *x*)⊆*F*(*y*)} is an *FWC*-subspace of (*X*, *D*; *φ*_*N*_) relative to the set {*u* ∈ *D* : *ξ*(*G*(*y*), *y*, *u*)⊆*W*(*y*)};
(iv) one of the following conditions holds:
  (iv_1_) *Y*∖*K*⊆⋃_*u*∈*N*_0__{*y* ∈ *Y* : *ξ*(*G*(*y*), *y*, *u*)⊆*W*(*y*)} for some *N*_0_ ∈ 〈*D*〉 and for each *N* = {*u*_0_, *u*_1_,…, *u*_*n*_}∈〈*D*〉, $\bar{T(\varphi_{N}(\Delta_{n}))}$ is a compact subset of *Y*;
  (iv_2_) for each *N* ∈ 〈*D*〉, there exists a subset *L*_*N*_ of *D* containing *N* such that *Q*(*L*_*N*_) is an *FWC*-subspace of (*X*, *D*; *φ*_*N*_) relative to *L*_*N*_, $\bar{\left(T\circ Q)(L_{N}\right)}$ is a compact subset of *Y*, and
    (60) $$\begin{array}{c} \bar{\left(T\circ Q)(L_{N}\right)}\setminus K\subseteq \underset{u\in L_{N}}{⋃}\left\{y\in Y:\xi \left(G\left(y\right),y,u\right)\subseteq W\left(y\right)\right\}. \end{array}$$

Then there exists $\hat{y}\in \bar{T(X)}{⋂}_{}K$ such that, for each *u* ∈ *D*, there exists $\bar{s}\in G(\hat{y})$ satisfying $\xi (\bar{s},\hat{y},u)\notin W(\hat{y})$.

**Proof**

Define two set-valued mappings *J* : *Y* × *X* → 2^*Z*^ and *L* : *Y* × *D* → 2^*Z*^ by

(61) J(y,x)=ζ(G(y),y,x)=⋃s∈G(y)ζ(s,y,x)for  each  (y,x)∈Y×X,L(y,u)=ξ(G(y),y,u)=⋃s∈G(y)ξ(s,y,u)for  each  (y,u)∈Y×D.

It is clear that all conditions of Theorem 20 are satisfied. By Theorem 20, there exists a point $\hat{y}\in \bar{T(X)}{⋂}_{}K$ such that $L(\hat{y},u)={⋃}_{s\in G(\hat{y})}\xi (s,\hat{y},u)⊈W(\hat{y})$ for each *u* ∈ *D*. Hence, for each *u* ∈ *D*, there exists $\bar{s}\in G(\hat{y})$ satisfying $\xi (\bar{s},\hat{y},u)\notin W(\hat{y})$. This completes the proof.

**Remark 25**

(1)Theorem 24 generalizes Theorem 4.4 of Fang and Huang [34] in the following aspects: (a) The underlying spaces of Theorem 18 and Theorem 4.4 of Fang and Huang [34] are *FWC*-spaces and *FC*-spaces, respectively. It follows from the previous analysis that *FWC*-spaces include *FC*-spaces as special cases; (b) the class $\tilde{ℬ}(X,D,Y)$ in Theorem 24 includes the class *ℬ*(*Y*, *X*) in Theorem 4.4 of Fang and Huang [33] as a special case; (c) (ii) Theorem 24 is weaker than (i) and (ii) of Theorem 4.4 of Fang and Huang [34]. In fact, by the proof of Theorem 4.4 of Fang and Huang [34], we can see that (i) and (ii) of Theorem 4.4 of Fang and Huang [34] imply (ii) of Theorem 24; (d) (iv_2_) of Theorem 24 is weaker than (vi) of Theorem 4.4 of Fang and Huang [34]. We point out that Theorem 24 also generalizes Theorem 3.2 and Corollary 3.2 of Lee and Kum [35] from topological vector spaces to *FWC*-spaces. We emphasis that *X*, *D*, *Z*, and *E* in Theorem 24 do not possess any linear, convex, and topological structure.

(2) Let *Z* and *E* in Theorem 24 be two topological spaces. Then by Theorem 4.4 of Fang and Huang [33], we can replace (ii) of Theorem 24 by the following conditions:

(ii)′ the graph of *W* is open in *Y* × *Z*;
(ii)′′ *G* is upper semicontinuous on each compact subset of *Y* with nonempty compact values and for each *u* ∈ *D*, *ξ*(·, ·, *u*) is continuous on each compact subset of *E* × *Y*.

**Remark 26**

The solution sets of generalized equilibrium problems considered in Theorems 18, 20, and 22–24 are compact subsets of $\bar{T(X)}{⋂}_{}K$. Indeed, by the proof of Theorems 6, 18, 20, and 22–24, we can see that these solution sets can be represented by $\left(\bar{T(X)}{⋂}_{}K){⋂}_{}({⋂}_{u\in D}(Y\setminus H^{-1}(u))\right)$, where *H* ^−1^ : *D* → 2^*Y*^ is a set-valued mapping with compactly open values. Thus, these solution sets are compactly closed subsets of the compact set $\bar{T(X)}{⋂}_{}K$. Therefore, the solution sets of generalized equilibrium problems considered in Theorems 18, 20, and 22–24 are compact subsets of $\bar{T(X)}{⋂}_{}K$.

## 5. Applications

Let *C* be a nonempty closed subset of a locally convex semireflexive topological vector space *X*, and let *F* be a real-valued function on *C* × *C*. In 1999, Isac et al. [36] first raised the open problem of finding $\hat{x}\in K$ such that $c_{1}\leq F(\hat{x},y)\leq c_{2}$ for each *y* ∈ *C*, where *c*_1_, *c*_2_ are two real numbers with *c*_1_ ≤ *c*_2_. Later, Li [37] introduced the concept of extremal subsets and then, by using the Fan-KKM theorem in topological vector spaces, he gave some positive answers to this open problem mentioned above. Recently, Fakhar and Zafarani [38] obtained an existence theorem of solutions to the equilibrium problems with lower and upper bounds under the setting of *G*-convex spaces.

In this section, we apply Theorem 18 to obtain existence results of solutions to the equilibrium problem with lower and upper bounds and minimax problem in *FWC*-spaces.

**Theorem 27**

Let (*X*, *D*; *φ*_*N*_) be an *FWC*-space and *K* a nonempty compact subset of a Hausdorff topological space *Y*. Let *Q* : *D* → 2^*X*^ and $T\in \tilde{ℬ}(X,D,Y)$ be set-valued mappings. Let *μ* and *ν* be real-valued functions on *Y* × *X* and *Y* × *D*, respectively. Let *g* and *h* be real-valued functions on *Y* such that *g*(*y*) ≤ *h*(*y*) for each *y* ∈ *Y*. Assume that

(i) for each *x* ∈ *X* and each *y* ∈ *T*(*x*), *g*(*y*) ≤ *μ*(*y*, *x*) ≤ *h*(*y*);
(ii) for each *u* ∈ *D*, the set {*y* ∈ *Y* : *g*(*y*) ≤ *ν*(*y*, *u*) ≤ *h*(*y*)} is compactly closed;
(iii) for each *y* ∈ *Y*, the set {*x* ∈ *X* : *μ*(*y*, *x*) > *h*(*y*)  *or* *μ*(*y*, *x*) < *g*(*y*)} is an *FWC*-subspace of (*X*, *D*; *φ*_*N*_) relative to the set {*u* ∈ *D* : *ν*(*y*, *u*) > *h*(*y*)  *or* *ν*(*y*, *u*) < *g*(*y*)};
(iv) one of the following conditions holds:
  (iv_1_) *Y*∖*K*⊆⋃_*u*∈*N*_0__{*y* ∈ *Y* : *ν*(*y*, *u*) > *h*(*y*)  *or* *ν*(*y*, *u*) < *g*(*y*)} for some *N*_0_ ∈ 〈*D*〉 and for each *N* = {*u*_0_, *u*_1_,…, *u*_*n*_}∈〈*D*〉, $\bar{T(\varphi_{N}(\Delta_{n}))}$ is a compact subset of *Y*;
  (iv_2_) for each *N* ∈ 〈*D*〉, there exists a subset *L*_*N*_ of *D* containing *N* such that *Q*(*L*_*N*_) is an *FWC*-subspace of (*X*, *D*; *φ*_*N*_) relative to *L*_*N*_, $\bar{\left(T\circ Q)(L_{N}\right)}$ is a compact subset of *Y*, and
    (62) (T∘Q)(LN)¯∖K ⊆⋃u∈LN{y∈Y:ν(y,u)>h(y)  or  ν(y,u)<g(y)}.

Then there exists $\hat{y}\in \bar{T(X)}{⋂}_{}K$ such that $g(\hat{y})\leq \nu (\hat{y},u)\leq h(\hat{y})$ for each *u* ∈ *D*.

**Proof**

Let *Z* = ℝ. Define three set-valued mappings *J* : *Y* × *X* → 2^*Z*^, *L* : *Y* × *D* → 2^*Z*^, *F* : *Y* → 2^*Z*^, and *W* : *Y* → 2^*Z*^ as follows:

(63) $$\begin{array}{c} J\left(y,x\right)=\left\{\mu \left(y,x\right)\right\}\;\text{for}\;\;\text{each}\;\;\left(y,x\right)\in Y\times X,\\ L\left(y,u\right)=\left\{\nu \left(y,u\right)\right\}\;\text{for}\;\;\text{each}\;\;\left(y,u\right)\in Y\times D,\\ F\left(y\right)=W\left(y\right)=\left[g\left(y\right),h\left(y\right)\right]\;\text{for}\;\;\text{each}\;\;y\in Y. \end{array}$$

It is clear that all conditions of Theorem 18 with *F* = *W* are satisfied. Therefore, by Theorem 18 with *F* = *W*, there exists a point $\hat{y}\in \bar{T(X)}{⋂}_{}K$ such that $L(\hat{y},u)\subseteq W(\hat{y})$ for each *u* ∈ *D*; that is, $g(\hat{y})\leq \nu (\hat{y},u)\leq h(\hat{y})$, for each *u* ∈ *D*. This completes the proof.

**Remark 28**

Theorem 27 generalizes Corollary 3.2 of Mitrović and Merkle [39] in the following aspects: (1) The underlying spaces of Theorem 27 and Corollary 3.2 in [39] are *FWC*-spaces and Hausdorff compact topological vector spaces, respectively. By the previous analysis, we know that *FWC*-spaces include Hausdorff compact topological vector spaces as special cases; (2) The condition that there are four functions in Theorem 27 is more general than the condition that there are three functions in Corollary 3.2 in [39]; (3) (ii) of Theorem 27 is weaker than (1) of Corollary 3.2 of Mitrović and Merkle [39]; (4) (iii) of Theorem 27 is weaker than (3) of of Corollary 3.2 of Mitrović and Merkle [39]. We point out that the proof of Theorem 27 is different from that of Corollary 3.2 of Mitrović and Merkle [39].

Let *g*(*y*) = *c*_1_ and *h*(*y*) = *c*_2_ for all *y* ∈ *Y*, where *c*_1_ and *c*_2_ are real numbers such that *c*_1_ ≤ *c*_2_. In this case, Theorem 27 deduces the following corollary.

**Corollary 29**

Let (*X*, *D*; *φ*_*N*_) be an *FWC*-space and *K* a nonempty compact subset of a Hausdorff topological space *Y*. Let *Q* : *D* → 2^*X*^ and $T\in \tilde{ℬ}(X,D,Y)$ be set-valued mappings. Let *μ* and *ν* be real-valued functions on *Y* × *X* and *Y* × *D*, respectively. Let *c*_1_ and *c*_2_ be two real numbers such that *c*_1_ ≤ *c*_2_. Assume that

(i) for each *x* ∈ *X* and each *y* ∈ *T*(*x*), *c*_1_ ≤ *μ*(*y*, *x*) ≤ *c*_2_;
(ii) for each *u* ∈ *D*, the set {*y* ∈ *Y* : *c*_1_ ≤ *ν*(*y*, *u*) ≤ *c*_2_} is compactly closed;
(iii) for each *y* ∈ *Y*, the set {*x* ∈ *X* : *μ*(*y*, *x*) > *c*_2_ *or* *μ*(*y*, *x*) < *c*_1_} is an *FWC*-subspace of (*X*, *D*; *φ*_*N*_) relative to the set {*u* ∈ *D* : *ν*(*y*, *u*) > *c*_2_ *or* *ν*(*y*, *u*) < *c*_1_};
(iv) one of the following conditions holds:
  (iv_1_) *Y*∖*K*⊆⋃_*u*∈*N*_0__{*y* ∈ *Y* : *ν*(*y*, *u*) > *c*_2_ *or* *ν*(*y*, *u*) < *c*_1_} for some *N*_0_ ∈ 〈*D*〉 and for each *N* = {*u*_0_, *u*_1_,…, *u*_*n*_}∈〈*D*〉, $\bar{T(\varphi_{N}(\Delta_{n}))}$ is a compact subset of *Y*;
  (iv_2_) for each *N* ∈ 〈*D*〉, there exists a subset *L*_*N*_ of *D* containing *N* such that *Q*(*L*_*N*_) is an *FWC*-subspace of (*X*, *D*; *φ*_*N*_) relative to *L*_*N*_, $\bar{\left(T\circ Q)(L_{N}\right)}$ is a compact subset of *Y*, and
    (64) (T∘Q)(LN)¯∖K ⊆⋃u∈LN{y∈Y:ν(y,u)>c2  or  ν(y,u)<c1}.

Then there exists $\hat{y}\in \bar{T(X)}{⋂}_{}K$ such that $c_{1}\leq \nu (\hat{y},u)\leq c_{2}$ for each *u* ∈ *D*.

Another special case of Corollary 29, stated below, is the case where *c*_1_ = *c*_2_ = *c*.

**Corollary 30**

Let (*X*, *D*; *φ*_*N*_) be an *FWC*-space and *K* a nonempty compact subset of a Hausdorff topological space *Y*. Let *Q* : *D* → 2^*X*^ and $T\in \tilde{ℬ}(X,D,Y)$ be set-valued mappings. Let *μ* and *ν* be real-valued functions on *Y* × *X* and *Y* × *D*, respectively. Let *c* be a real number. Assume that

(i) for each *x* ∈ *X* and each *y* ∈ *T*(*x*), *μ*(*y*, *x*) = *c*;
(ii) for each *u* ∈ *D*, the set {*y* ∈ *Y* : *ν*(*y*, *u*) = *c*} is compactly closed;
(iii) for each *y* ∈ *Y*, the set {*x* ∈ *X* : *μ*(*y*, *x*) > *c* *or* *μ*(*y*, *x*) < *c*} is an *FWC*-subspace of (*X*, *D*; *φ*_*N*_) relative to the set {*u* ∈ *D* : *ν*(*y*, *u*) > *c* *or* *ν*(*y*, *u*) < *c*};
(iv) one of the following conditions holds:
  (iv_1_) *Y*∖*K*⊆⋃_*u*∈*N*_0__{*y* ∈ *Y* : *ν*(*y*, *u*) > *c* *or* *ν*(*y*, *u*) < *c*} for some *N*_0_ ∈ 〈*D*〉 and for each *N* = {*u*_0_, *u*_1_,…, *u*_*n*_}∈〈*D*〉, $\bar{T(\varphi_{N}(\Delta_{n}))}$ is a compact subset of *Y*;
  (iv_2_) for each *N* ∈ 〈*D*〉, there exists a subset *L*_*N*_ of *D* containing *N* such that *Q*(*L*_*N*_) is an *FWC*-subspace of (*X*, *D*; *φ*_*N*_) relative to *L*_*N*_, $\bar{\left(T\circ Q)(L_{N}\right)}$ is a compact subset of *Y*, and
    (65) (T∘Q)(LN)¯∖K ⊆⋃u∈LN{y∈Y:ν(y,u)>c  or  ν(y,u)<c}.

Then there exists $\hat{y}\in \bar{T(X)}{⋂}_{}K$ such that $\nu (\hat{y},u)=c$ for each *u* ∈ *D*.

**Remark 31**

It is interesting to compare Corollary 30 with Corollary 3.1 of Li [37] in the following aspects: (1) the underlying spaces in Corollary 30 are *FWC*-spaces without any linear, convex, and topological structure, which include the corresponding underlying spaces (i.e, Hausdorff topological vector spaces) in Corollary 3.1 of Li [37] as special cases; (2) (i) of Corollary 30 is weaker than (i) of Corollary 3.1 of Li [37]; (3) (ii) of Corollary 30 is weaker than (iv) of Corollary 3.1 of Li [37]; (4) (iii) of Corollary 30 is weaker than (ii) of Corollary 3.1 of Li [37]; (5) (iv) of Corollary 30 is neither stronger nor weaker than (iii) of Corollary 3.1 of Li [37].

As a consequence of Corollary 29, we can obtain the following corollary, which improves and generalizes Theorems 2.3-2.4 of Verma [20], Theorem 2.6 of Verma [21], and Corollary 3.4 of Fakhar and Zafarani [40].

**Corollary 32**

Let (*X*, *D*; *φ*_*N*_) be an *FWC*-space and *K* a nonempty compact subset of a Hausdorff topological space *Y*. Let *Q* : *D* → 2^*X*^ and $T\in \tilde{ℬ}(X,D,Y)$ be set-valued mappings. Let *μ*_1_ and *ν*_1_ be real-valued functions on *Y* × *X* and *Y* × *D*, respectively. Let *c* be a real number. Assume that

(i) for each *x* ∈ *X* and each *y* ∈ *T*(*x*), *μ*_1_(*y*, *x*) ≤ *c*;
(ii) for each *u* ∈ *D*, the set {*y* ∈ *Y* : *ν*_1_(*y*, *u*) ≤ *c*} is compactly closed;
(iii) for each *y* ∈ *Y*, the set {*x* ∈ *X* : *μ*_1_(*y*, *x*) > *c*} is an *FWC*-subspace of (*X*, *D*; *φ*_*N*_) relative to the set {*u* ∈ *D* : *ν*_1_(*y*, *u*) > *c*};
(iv) one of the following conditions holds:
  (iv_1_) *Y*∖*K*⊆⋃_*u*∈*N*_0__{*y* ∈ *Y* : *ν*_1_(*y*, *u*) > *c*} for some *N*_0_ ∈ 〈*D*〉 and for each *N* = {*u*_0_, *u*_1_,…, *u*_*n*_}∈〈*D*〉, $\bar{T(\varphi_{N}(\Delta_{n}))}$ is a compact subset of *Y*;
  (iv_2_) for each *N* ∈ 〈*D*〉, there exists a subset *L*_*N*_ of *D* containing *N* such that *Q*(*L*_*N*_) is an *FWC*-subspace of (*X*, *D*; *φ*_*N*_) relative to *L*_*N*_, $\bar{\left(T\circ Q)(L_{N}\right)}$ is a compact subset of *Y*, and
    (66) $$\begin{array}{c} \bar{\left(T\circ Q)(L_{N}\right)}\setminus K\subseteq \underset{u\in L_{N}}{⋃}\left\{y\in Y:\nu_{1}\left(y,u\right)>c\right\}. \end{array}$$

Then there exists $\hat{y}\in \bar{T(X)}{⋂}_{}K$ such that $\nu_{1}(\hat{y},u)\leq c$ for each *u* ∈ *D*.

**Proof**

Define real-valued functions *μ* : *Y* × *X* → ℝ and *ν* : *Y* × *D* → ℝ by

(67) $$\begin{array}{c} \mu \left(y,x\right)=e^{\mu_{1}(y,x)}\;\text{for}\;\;\text{each}\;\;\left(y,x\right)\in Y\times X,\\ \nu \left(y,u\right)=e^{\nu_{1}(y,u)}\;\text{for}\;\;\text{each}\;\;\left(y,u\right)\in Y\times D. \end{array}$$

Taking *c*_1_ = 0 and *c*_2_ = *e* ^*c*^ in Corollary 29, we can see that all conditions of Corollary 29 are satisfied for *μ* and *ν*. Therefore, by Corollary 29, there exists a point $\hat{y}\in \bar{T(X)}{⋂}_{}K$ such that $0\leq \nu (\hat{y},u)\leq e^{c}$ for each *u* ∈ *D*; that is, $\nu_{1}(\hat{y},u)\leq c$ for each *u* ∈ *D*. This completes the proof.

**Remark 33**

(ii)-(iii) of Corollary 32 can be replaced by the following conditions, respectively.

(ii)′ For every *u* ∈ *D*, *ν*_1_(·, *u*) is lower semicontmuous on each nonempty compact subset of *Y*.
(iii)′ For every *N* = {*u*_0_, *u*_1_,…, *u*_*n*_}∈〈*D*〉, every {*u*_*i*_0__, *u*_*i*_1__,…, *u*_*i*_*k*__}⊆*N*, and every *y* ∈ *Y*, we have *μ*_1_(*y*, *x*) ≥ min⁡_0≤*j*≤*k*_ *ν*_1_(*y*, *u*_*i*_*j*__) for all *x* ∈ *φ*_*N*_(Δ_*k*_).

It is clear that (ii)′ implies (ii) of Corollary 32. Now, we show that (iii)′ implies (iii) of Corollary 32. In fact, if (iii) of Corollary 32 does not hold, then there exist *y* ∈ *Y*, *N* = {*u*_0_, *u*_1_,…, *u*_*n*_}∈〈*D*〉, and {*u*_*i*_0__, *u*_*i*_1__,…, *u*_*i*_*k*__}⊆*N*∩{*u* ∈ *D* : *ν*_1_(*y*, *u*) > *c*} such that *φ*_*N*_(Δ_*k*_)⊈{*x* ∈ *X* : *μ*_1_(*y*, *x*) > *c*}. Hence, there exists *x* ∈ *φ*_*N*_(Δ_*k*_) such that *μ*_1_(*y*, *x*) ≤ *c*. Since {*u*_*i*_0__, *u*_*i*_1__,…, *u*_*i*_*k*__}⊆*N*∩{*u* ∈ *D* : *ν*_1_(*y*, *u*) > *c*}, we have *ν*_1_(*y*, *u*_*i*_*j*__) > *c* for each *j* ∈ {0,1,…, *k*}. By (iii)′, we obtain the following contradiction:

(68) $$\begin{array}{c} c\geq \mu_{1}\left(y,x\right)\geq \underset{0\leq j\leq k}{\min}\nu_{1}\left(y,u_{i_{j}}\right)>c. \end{array}$$

Therefore, (iii) of Corollary 32 must hold.

**Remark 34**

Theorem 6 is equivalent to Corollary 32. We first show that Theorem 6 implies that Corollary 32. Define *P* : *X* → 2^*Y*^ and *H* : *Y* → 2^*D*^ by

(69) $$\begin{array}{c} P\left(x\right)=\left\{y\in Y:\mu_{1}\left(y,x\right)\leq c\right\}\;\text{for}\;\;\text{each}\;\;x\in X,\\ H\left(y\right)=\left\{u\in D:\nu_{1}\left(y,u\right)>c\right\}\;\text{for}\;\;\text{each}\;\;u\in D. \end{array}$$

By using the same method as in Theorem 18, we know that (iii) of Theorem 6 holds. We can see that the other conditions of Theorem 6 are satisfied. Therefore, by Theorem 6, there exists a point $\hat{y}\in \bar{T(X)}{⋂}_{}K$ such that $H(\hat{y})=\emptyset$, which implies that $\nu_{1}(\hat{y},u)\leq c$ for each *u* ∈ *D*.

Conversely, let *c* ∈ ℝ be given. Let us define two real-valued functions *μ*_1_ : *Y* × *X* → ℝ and *ν*_1_ : *Y* × *D* → ℝ by

(70) $$\begin{array}{c} \mu_{1}\left(y,x\right)=\{\begin{array}{cc} c, & y\in P\left(x\right),\\ c+1, & y\notin P\left(x\right), \end{array}\\ \nu_{1}\left(y,u\right)=\{\begin{array}{cc} c, & y\notin H^{-1}\left(u\right),\\ c+1, & y\in H^{-1}\left(u\right). \end{array} \end{array}$$

We can see that *μ*_1_ and *ν*_1_ satisfy all conditions of Corollary 32. Therefore, by Corollary 32, there exists a point $\hat{y}\in \bar{T(X)}{⋂}_{}K$ such that $\nu_{1}(\hat{y},u)\leq c$ for each *u* ∈ *D*; that is, $\hat{y}\notin H^{-1}(u)$ for each *u* ∈ *D*, which implies that $H(\hat{y})=\emptyset$.

**Corollary 35**

Let (*X*, *D*; *φ*_*N*_), *Y* be as in Theorem 27, and let $T\in \tilde{ℬ}(X,D,Y)$ be a compact set-valued mapping. Let *μ*_1_ and *ν*_1_ be real-valued functions on *Y* × *X* and *Y* × *D*, respectively. Let *c* be a real number. Assume that

- for each *x* ∈ *X* and each *y* ∈ *T*(*x*), *μ*_1_(*y*, *x*) ≤ *c*;
- for each *u* ∈ *D*, the set {*y* ∈ *Y* : *ν*_1_(*y*, *u*) ≤ *c*} is compactly closed;
- for each *y* ∈ *Y*, the set {*x* ∈ *X* : *μ*_1_(*y*, *x*) > *c*} is an *FWC*-subspace of (*X*, *D*; *φ*_*N*_) relative to the set {*u* ∈ *D* : *ν*_1_(*y*, *u*) > *c*}.

Then there exists $\hat{y}\in \bar{T(X)}$ such that $\nu_{1}(\hat{y},u)\leq c$ for each *u* ∈ *D*.

**Proof**

Define a set-valued mapping *Q* : *D* → 2^*X*^ by *Q*(*u*) = *X* for each *u* ∈ *D*. For each *N* ∈ 〈*D*〉, let *L*_*N*_ = *D*. Let $K=\bar{T(X)}$. Then (iv_2_) of Corollary 32 is satisfied automatically. Therefore, the conclusion of Corollary 35 follows from Corollary 32.

**Remark 36**

Corollary 35 generalizes Theorem 3.3 of Tan [41] in the following aspects: (1) The underlying spaces of Corollary 35 are *FWC*-spaces which contain* G-convex* spaces adopted in Theorem 3.3 of Tan [41]; (2) There are two functions in Corollary 35, but there is only one function in Theorem 3.3 of Tan [41]; (3) the condition that each *G*-co(*A*) is compact in Theorem 3.3 of Tan [41] is dropped; (4) (ii) of Corollary 35 is weaker than (1) of Theorem 3.3 of Tan [41]. In fact, the lower semicontinuity of a function implies (ii) of Corollary 35; (5) combining Proposition 2.1 of Tan [41] and Remark 2.1, we can see that (iii) of Corollary 35 is weaker than (2) of Theorem 3.3 of Tan [41]. Corollary 35 also generalizes Theorem 3.1 of Zeng et al. [33] from topological vector spaces to *FWC*-spaces without any linear, convex, and topological structure. The comparison details between Corollary 35 and Theorem 3.1 of Zeng et al. [33] are left up to the reader to finish.

**Theorem 37**

Let (*X*, *D*; *φ*_*N*_) be an *FWC*-space and *K* a nonempty compact subset of a Hausdorff topological space *Y*. Let *Q* : *D* → 2^*X*^ and $T\in \tilde{ℬ}(X,D,Y)$ be set-valued mappings. Let *μ*_1_ and *ν*_1_ be real-valued functions on *Y* × *X* and *Y* × *D*, respectively. Let *c* be a real number. Assume that

(i) for each *u* ∈ *D*, the set {*y* ∈ *Y* : *ν*_1_(*y*, *u*) ≤ *c*} is compactly closed;
(ii) for each *y* ∈ *Y*, the set {*x* ∈ *X* : *μ*_1_(*y*, *x*) > *c*} is an *FWC*-subspace of (*X*, *D*; *φ*_*N*_) relative to the set {*u* ∈ *D* : *ν*_1_(*y*, *u*) > *c*};
(iii) one of the following conditions holds:
  (iii_1_) *Y*∖*K*⊆⋃_*u*∈*N*_0__{*y* ∈ *Y* : *ν*_1_(*y*, *u*) > *c*} for some *N*_0_ ∈ 〈*D*〉 and for each *N* = {*u*_0_, *u*_1_,…, *u*_*n*_}∈〈*D*〉, $\bar{T(\varphi_{N}(\Delta_{n}))}$ is a compact subset of *Y*;
  (iii_2_) for each *N* ∈ 〈*D*〉, there exists a subset *L*_*N*_ of *D* containing *N* such that *Q*(*L*_*N*_) is an *FWC*-subspace of (*X*, *D*; *φ*_*N*_) relative to *L*_*N*_, $\bar{\left(T\circ Q)(L_{N}\right)}$ is a compact subset of *Y*, and
    (71) $$\begin{array}{c} \bar{\left(T\circ Q)(L_{N}\right)}\setminus K\subseteq \underset{u\in L_{N}}{⋃}\left\{y\in Y:\nu_{1}\left(y,u\right)>c\right\}. \end{array}$$

Then we have the following alternatives:

- there exist $\hat{x}\in X$ and $\hat{y}\in T(\hat{x})$ such that $\mu_{1}(\hat{y},\hat{x})>c$;
- there exists $\hat{y}\in \bar{T(X)}{⋂}_{}K$ such that $\nu_{1}(\hat{y},u)\leq c$ for each *u* ∈ *D*.

**Proof**

If (a) is false, then it follows that for each *x* ∈ *X* and each *y* ∈ *T*(*x*), *μ*_1_(*y*, *x*) ≤ *c*. Hence, by Corollary 32, there exists a point $\hat{y}\in \bar{T(X)}{⋂}_{}K$ such that $\nu_{1}(\hat{y},u)\leq c$ for each *u* ∈ *D*. This completes the proof.

**Theorem 38**

Let (*X*, *D*; *φ*_*N*_) be an *FWC*-space and *K* a nonempty compact subset of a Hausdorff topological space *Y*. Let *Q* : *D* → 2^*X*^ and $T\in \tilde{ℬ}(X,D,Y)$ be set-valued mappings. Let *μ*_1_ and *ν*_1_ be real-valued functions on *Y* × *X* and *Y* × *D*, respectively. Assume that sup⁡_*x*∈*X*,*y*∈*T*(*x*)_ *μ*_1_(*y*, *x*)<+*∞* and the following conditions hold:

(i) for each *u* ∈ *D*, the set {*y* ∈ *Y* : *ν*_1_(*y*, *u*) ≤ sup⁡_*x*∈*X*,*y*∈*T*(*x*)_ *μ*_1_(*y*, *x*)} is compactly closed;
(ii) for each *y* ∈ *Y*, the set {*x* ∈ *X* : *μ*_1_(*y*, *x*) > sup⁡_*x*∈*X*,*y*∈*T*(*x*)_ *μ*_1_(*y*, *x*)} is an *FWC*-subspace of (*X*, *D*; *φ*_*N*_) relative to the set {*u* ∈ *D* : *ν*_1_(*y*, *u*) > sup⁡_*x*∈*X*,*y*∈*T*(*x*)_ *μ*_1_(*y*, *x*)};
(iii) either
  (iii_1_) *Y*∖*K*⊆⋃_*u*∈*N*_0__{*y* ∈ *Y* : *ν*_1_(*y*, *u*) > sup⁡_*x*∈*X*,*y*∈*T*(*x*)_ *μ*_1_(*y*, *x*)} for some *N*_0_ ∈ 〈*D*〉 and for each *N* = {*u*_0_, *u*_1_,…, *u*_*n*_}∈〈*D*〉, $\bar{T(\varphi_{N}(\Delta_{n}))}$ is a compact subset of *Y* or
  (iii_2_) for each *N* ∈ 〈*D*〉, there exists a subset *L*_*N*_ of *D* containing *N* such that *Q*(*L*_*N*_) is an *FWC*-subspace of (*X*, *D*; *φ*_*N*_) relative to *L*_*N*_, $\bar{\left(T\circ Q)(L_{N}\right)}$ is a compact subset of *Y*, and
    (72) (T∘Q)(LN)¯∖K  ⋃u∈LN{y∈Y:ν1(y,u)>sup⁡x∈X,y∈T(x)μ1(y,x)}.

Then there exists $\hat{y}\in \bar{T(X)}{⋂}_{}K$ such that $\nu_{1}(\hat{y},u)\leq \sup_{x\in X,y\in T(x)}\mu_{1}(y,x)$ for each *u* ∈ *D*. In particular, we have $\inf_{y\in K\cap \bar{T(X)}}\sup_{u\in D}\nu_{1}(y,u)\leq \sup_{x\in X,y\in T(x)}\mu_{1}(y,x)$.

**Proof**

Let *c* = sup⁡_*x*∈*X*,*y*∈*T*(*x*)_ *μ*_1_(*y*, *x*). By the definition of *c*, (a) of Theorem 37 does not hold. Hence, (b) of Theorem 37 is satisfied. So, there exists a point $\hat{y}\in \bar{T(X)}{⋂}_{}K$ such that $\nu_{1}(\hat{y},u)\leq c$ for each *u* ∈ *D*. In particular, we have $\inf_{y\in K\cap \bar{T(X)}}\sup_{u\in D}\nu_{1}(y,u)\leq \sup_{x\in X,y\in T(x)}\mu_{1}(y,x)$. This completes the proof.

**Remark 39**

By setting *μ*_1_′ = −*μ*_1_ and *ν*_1_′ = −*ν*_1_ and adjusting the corresponding conditions of Theorem 38, we know that Theorem 38 can be restated with the conclusion that there exists a point $\hat{y}\in \bar{T(X)}{⋂}_{}K$ such that $\nu_{1}^{\prime }(\hat{y},u)\geq \inf_{x\in X,y\in T(x)}\mu_{1}^{\prime }(y,x)$ for each *u* ∈ *D*. In particular, we have $\sup_{y\in K\cap \bar{T(X)}}\inf_{u\in D}\nu_{1}^{\prime }(y,u)\geq \inf_{x\in X,y\in T(x)}\mu_{1}^{\prime }(y,x)$. Thus, Theorem 38 generalizes Theorem 3 of Yuan [28] from Hausdorff topological vector spaces to *FWC*-spaces.

If *T* in Theorem 38 is a single-valued mapping, then we have the following corollary.

**Corollary 40**

Let (*X*, *D*; *φ*_*N*_), *K*, *Y*, and *Q* be as in Theorem 27. Let $T\in \tilde{ℬ}(Y,D,X)$ be a single-valued mapping. Let *μ*_1_ and *ν*_1_ be real-valued functions on *Y* × *X* and *Y* × *D*, respectively. Assume that sup⁡_*x*∈*X*_ *μ*_1_(*T*(*x*), *x*)<+*∞* and the following conditions hold:

(i) for each *u* ∈ *D*, the set {*y* ∈ *Y* : *ν*_1_(*y*, *u*) ≤ sup⁡_*x*∈*X*_ *μ*_1_(*T*(*x*), *x*)} is compactly closed;
(ii) for each *y* ∈ *Y*, the set {*x* ∈ *X* : *μ*_1_(*y*, *x*) > sup⁡_*x*∈*X*_ *μ*_1_(*T*(*x*), *x*)} is an *FWC*-subspace of (*X*, *D*; *φ*_*N*_) relative to the set {*u* ∈ *D* : *ν*_1_(*y*, *u*) > sup⁡_*x*∈*X*_ *μ*_1_(*T*(*x*), *x*)};
(iii) either
  (iii_1_) *Y*∖*K*⊆⋃_*u*∈*N*_0__{*y* ∈ *Y* : *ν*_1_(*y*, *u*) > sup⁡_*x*∈*X*_ *μ*_1_(*T*(*x*), *x*)} for some *N*_0_ ∈ 〈*D*〉 and for each *N* = {*u*_0_, *u*_1_,…, *u*_*n*_}∈〈*D*〉, $\bar{T(\varphi_{N}(\Delta_{n}))}$ is a compact subset of *Y* or
  (iii_2_) for each *N* ∈ 〈*D*〉, there exists a subset *L*_*N*_ of *D* containing *N* such that *Q*(*L*_*N*_) is an *FWC*-subspace of (*X*, *D*; *φ*_*N*_) relative to *L*_*N*_, $\bar{\left(T\circ Q)(L_{N}\right)}$ is a compact subset of *Y*, and
    (73) (T∘Q)(LN)¯∖K ⊆⋃u∈LN{y∈Y:ν1(y,u)>sup⁡x∈Xμ1(T(x),x)}.

Then there exists $\hat{y}\in \bar{T(X)}{⋂}_{}K$ such that $\nu_{1}(\hat{y},u)\leq \sup_{x\in X}\mu_{1}(T(x),x)$ for each *u* ∈ *D*. In particular, we have $\inf_{y\in K\cap \bar{T(X)}}\sup_{u\in D}\nu_{1}(y,u)\leq \sup_{x\in X}\mu_{1}(T(x),x)$.

By taking *X* = *Y* and *T*(*x*) = {*x*} for all *x* ∈ *X*, we can obtain the following result from Corollary 40.

**Corollary 41**

Let (*X*, *D*; *φ*_*N*_) be an *FWC*-space, where *X* is a Hausdorff topological space. Let *K* be a nonempty compact subset of *X*. Let *Q* : *D* → 2^*X*^ be a set-valued mapping and let $I_{X}\in \tilde{ℬ}(X,D,X)$, where *I*_*X*_ is the identity mapping on *X*. Let *μ*_1_ and *ν*_1_ be real-valued functions on *X* × *X* and *X* × *D*, respectively. Assume that sup⁡_*x*∈*X*_ *μ*_1_(*x*, *x*)<+*∞* and the following conditions hold:

(i) for each *u* ∈ *D*, the set {*y* ∈ *X* : *ν*_1_(*y*, *u*) ≤ sup⁡_*x*∈*X*_ *μ*_1_(*x*, *x*)} is compactly closed;
(ii) for each *y* ∈ *X*, the set {*x* ∈ *X* : *μ*_1_(*y*, *x*) > sup⁡_*x*∈*X*_ *μ*_1_(*x*, *x*)} is an *FWC*-subspace of (*X*, *D*; *φ*_*N*_) relative to the set {*u* ∈ *D* : *ν*_1_(*y*, *u*) > sup⁡_*x*∈*X*_ *μ*_1_(*x*, *x*)};
(iii) either
  (iii_1_) *X*∖*K*⊆⋃_*u*∈*N*_0__{*y* ∈ *X* : *ν*_1_(*y*, *u*) > sup⁡_*x*∈*X*_ *μ*_1_(*x*, *x*)} for some *N*_0_ ∈ 〈*D*〉 and *φ*_*N*_ : Δ_*n*_ → 2^*X*^ is a compact set-valued mapping for each *N* = {*u*_0_, *u*_1_,…, *u*_*n*_}∈〈*D*〉 or
  (iii_2_) for each *N* ∈ 〈*D*〉, there exists a subset *L*_*N*_ of *D* containing *N* such that $\bar{Q(L_{N})}$ is a compact *FWC*-subspace of (*X*, *D*; *φ*_*N*_) relative to *L*_*N*_ and
    (74) $$\begin{array}{c} \bar{Q(L_{N})}\setminus K\subseteq \underset{u\in L_{N}}{⋃}\left\{y\in X:\nu_{1}\left(y,u\right)>\underset{x\in X}{\sup}\mu_{1}\left(x,x\right)\right\}. \end{array}$$

Then there exists $\hat{y}\in K$ such that $\nu_{1}(\hat{y},u)\leq \sup_{x\in X}\mu_{1}(x,x)$ for each *u* ∈ *D*. In particular, we have inf⁡_*y*∈*K*_sup⁡_*u*∈*D*_ *ν*_1_(*y*, *u*) ≤ sup⁡_*x*∈*X*_ *μ*_1_(*x*, *x*).

**Remark 42**

Corollary 41 generalizes Corollary 5 of Jin and Cheng [42] in the following aspects: (1) The underlying spaces of Corollary 41 are *FWC*-spaces which include* L-*convex spaces adopted in Corollary 5 of Jin and Cheng [42] as special cases; (2) the condition that each *H*(*A*) in Corollary 5 of Jin and Cheng [42] is compact is dropped; (3) (i) of Corollary 41 is weaker than (i) of Corollary 5 of Jin and Cheng [42]. In fact, the condition that a function is lower semicontinuous on compact subset of *X* implies (i) of Corollary 41; (4) it is easy to verify that (ii) of Corollary 41 is weaker than (ii) of Corollary 5 of Jin and Cheng [42]; (5) (iii) of Corollary 41 is weaker than (iii) of Corollary 5 of Jin and Cheng [42]. Additionally, Corollary 41 is quite differen7t from Theorem 1 of Kim [43] because the underlying spaces of Theorem 1 of Kim [43] are Hausdorff topological vector spaces and the conditions of Theorem 1 of Kim [43] are different from that of Corollary 41.

**Corollary 43**

Let (*X*, *D*; *φ*_*N*_) be an *FWC*-space, where *X* is a Hausdorff topological space. Let *K* be a nonempty compact subset of *X*. Let *Q* : *D* → 2^*X*^ be a set-valued mapping and let $I_{X}\in \tilde{ℬ}(X,D,X)$, where *I*_*X*_ is the identity mapping on *X*. Let *μ*_1_ and *ν*_1_ be real-valued functions on *X* × *X* and *X* × *D*, respectively. Assume that inf⁡_*x*∈*X*_ *μ*_1_(*x*, *x*)>−*∞* and the following conditions hold:

(i) for each *u* ∈ *D*, the set {*y* ∈ *X* : *ν*_1_(*y*, *u*) ≥ inf⁡_*x*∈*X*_ *μ*_1_(*x*, *x*)} is compactly closed;
(ii) for each *y* ∈ *X*, the set {*x* ∈ *X* : *μ*_1_(*y*, *x*) < inf⁡_*x*∈*X*_ *μ*_1_(*x*, *x*)} is an *FWC*-subspace of (*X*, *D*; *φ*_*N*_) relative to the set {*u* ∈ *D* : *ν*_1_(*y*, *u*) < inf⁡_*x*∈*X*_ *μ*_1_(*x*, *x*)};
(iii) either
  (iii_1_) *X*∖*K*⊆⋃_*u*∈*N*_0__{*y* ∈ *X* : *ν*_1_(*y*, *u*) < inf⁡_*x*∈*X*_ *μ*_1_(*x*, *x*)} for some *N*_0_ ∈ 〈*D*〉 and *φ*_*N*_ : Δ_*n*_ → 2^*X*^ is a compact set-valued mapping for each *N* = {*u*_0_, *u*_1_,…, *u*_*n*_}∈〈*D*〉 or
  (iii_2_) for each *N* ∈ 〈*D*〉, there exists a subset *L*_*N*_ of *D* containing *N* such that $\bar{Q(L_{N})}$ is a compact *FWC*-subspace of (*X*, *D*; *φ*_*N*_) relative to *L*_*N*_ and
    (75) $$\begin{array}{c} \bar{Q(L_{N})}\setminus K\subseteq \underset{u\in L_{N}}{⋂}\left\{y\in X:\nu_{1}\left(y,u\right)<\underset{x\in X}{\inf}\mu_{1}\left(x,x\right)\right\}. \end{array}$$

Then there exists $\hat{y}\in K$ such that $\nu_{1}(\hat{y},u)\geq \inf_{x\in X}\mu_{1}(x,x)$ for each *u* ∈ *D*. In particular, we have sup⁡_*y*∈*K*_inf⁡_*u*∈*D*_ *ν*_1_(*y*, *u*) ≥ inf⁡_*x*∈*X*_ *μ*_1_(*x*, *x*).

**Proof**

By setting *μ*_1_′ = −*μ*_1_ and *ν*_1_′ = −*ν*_1_, we can see that the conclusion of Corollary 43 holds from Corollary 41.

**Theorem 44**

Let (*Y*; *φ*_*N*_1__^1^) and (*X*; *φ*_*N*_2__^2^) be two *FWC*-spaces, where *X* and *Y* are two Hausdorff topological spaces. Let $T\in \tilde{ℬ}(Y\times X,Y\times X)$ be a compact set-valued mapping, where (*Y* × *X*; *φ*_*N*_1__^1^ × *φ*_*N*_2__^2^) is an *FWC*-space defined as in Lemma 5. Let *μ*_1_ be a real-valued function on *Y* × *X*. Assume that

- for each (*z*, *w*) ∈ *Y* × *X*, each (*y*, *x*) ∈ *T*(*z*, *w*), and each *α* ∈ ℝ, *μ*_1_(*z*, *x*) ≤ *α* or *μ*_1_(*y*, *w*) ≥ *α*;
- for each (*z*, *w*) ∈ *Y* × *X* and each *α* ∈ ℝ, the sets {*x* ∈ *X* : *μ*_1_(*z*, *x*) ≤ *α*} and {*y* ∈ *Y* : *μ*_1_(*y*, *w*) ≥ *α*} are compactly closed;
- for each *x* ∈ *X* and each *α* ∈ ℝ, the set {*z* ∈ *Y* : *μ*_1_(*z*, *x*) > *α*} is an *FWC*-subspace of (*Y*; *φ*_*N*_1__^1^);
- for each *y* ∈ *Y* and each *α* ∈ ℝ, the set {*w* ∈ *X* : *μ*_1_(*y*, *w*) < *α*} is an *FWC*-subspace of (*X*; *φ*_*N*_2__^2^).

Then inf⁡_*x*∈*X*_sup⁡_*y*∈*Y*_ *μ*_1_(*y*, *x*) = sup⁡_*y*∈*Y*_inf⁡_*x*∈*X*_ *μ*_1_(*y*, *x*).

**Proof**

It is clear that the following inequality

(76) $$\begin{array}{c} \underset{x\in X}{\inf}\;\underset{y\in Y}{\sup}\mu_{1}\left(y,x\right)\geq \underset{y\in Y}{\sup}\;\underset{x\in X}{\inf}\mu_{1}\left(y,x\right) \end{array}$$

is always true. In order to prove that the equality holds, it suffices to show the following inequality:

(77) $$\begin{array}{c} \underset{x\in X}{\inf}\;\underset{y\in Y}{\sup}\mu_{1}\left(y,x\right)\leq \underset{y\in Y}{\sup}\;\underset{x\in X}{\inf}\mu_{1}\left(y,x\right). \end{array}$$

Suppose the contrary. Then we have

(78) $$\begin{array}{c} \underset{x\in X}{\inf}\;\underset{y\in Y}{\sup}\mu_{1}\left(y,x\right)>\underset{y\in Y}{\sup}\;\underset{x\in X}{\inf}\mu_{1}\left(y,x\right). \end{array}$$

It follows that there exists *α* ∈ ℝ such that

(79) $$\begin{array}{c} \underset{x\in X}{\inf}\;\underset{y\in Y}{\sup}\mu_{1}\left(y,x\right)>\alpha >\underset{y\in Y}{\sup}\;\underset{x\in X}{\inf}\mu_{1}\left(y,x\right), \end{array}$$

which shows that for each (*y*, *x*) ∈ *Y* × *X*, there exists $(\bar{y},\bar{x})\in Y\times X$ such that

(80) $$\begin{array}{c} \mu_{1}\left(\bar{y},x\right)>\alpha ,\;\;\mu_{1}\left(y,\bar{x}\right)<\alpha . \end{array}$$

Following the method in the proof of Theorem 4.4 of Tan [41], we define the real-valued function *υ* : (*Y* × *X*)×(*Y* × *X*) → ℝ by

(81) $$\begin{array}{c} \upsilon \left(\left(y,x\right),\left(z,w\right)\right)=\{\begin{array}{cc} 1, & \text{if}\;\;\mu_{1}\left(z,x\right)>\alpha ,\\ & \mu_{1}\left(y,w\right)<\alpha ,\\ 0, & \text{otherwise}. \end{array} \end{array}$$

By (i) and the definition of *υ*, for each (*z*, *w*) ∈ *Y* × *X*, each (*y*, *x*) ∈ *T*(*z*, *w*), we have *υ*((*y*, *x*), (*z*, *w*)) ≤ 0. For each (*z*, *w*) ∈ *Y* × *X*, we have

(82) {(y,x)∈Y×X:υ((y,x),(z,w))≤0} =(Y×{x∈X:μ1(z,x)≤α}) ⋃({y∈Y:μ1(y,w)≥α}×X).

Then by (ii), we know that for each (*z*, *w*) ∈ *Y* × *X*, the set {(*y*, *x*) ∈ *Y* × *X* : *υ*((*y*, *x*), (*z*, *w*)) ≤ 0} is compactly closed.

Now, we show that for each (*y*, *x*) ∈ *Y* × *X*, the set {(*z*, *w*) ∈ *Y* × *X* : *υ*((*y*, *x*), (*z*, *w*)) > 0} is an *FWC*-space of (*Y* × *X*; *φ*_*N*_1__^1^ × *φ*_*N*_2__^2^). In fact, for each (*y*, *x*) ∈ *Y* × *X*, we have the following:

(83) {(z,w)∈Y×X:υ((y,x),(z,w))>0} =({z∈Y:μ1(z,x)>α}×X) ⋂(Y×{w∈X:μ1(y,w)<α}).

Then by (iii) and (iv), for each *N* = *N*_1_ × *N*_2_ = {(*z*_0_, *w*_0_), (*z*_1_, *w*_1_),…, (*z*_*n*_, *w*_*n*_)}∈〈*Y* × *X*〉 and each {(*z*_*i*_0__, *w*_*i*_0__), (*z*_*i*_1__, *w*_*i*_1__),…, (*z*_*i*_*k*__, *w*_*i*_*k*__)}⊆{(*z*, *w*) ∈ *Y* × *X* : *υ*((*y*, *x*), (*z*, *w*)) > 0}∩*N*, we have

(84) $$\begin{array}{c} \varphi_{N_{1}}^{1}\left(\Delta_{k}\right)\subseteq \left\{z\in Y:\mu_{1}\left(z,x\right)>\alpha \right\},\\ \varphi_{N_{2}}^{2}\left(\Delta_{k}\right)\subseteq \left\{w\in X:\mu_{1}\left(y,w\right)<\alpha \right\}. \end{array}$$

Since *φ*_*N*_(Δ_*k*_) = *φ*_*N*_1__^1^(Δ_*k*_) × *φ*_*N*_2__^2^(Δ_*k*_), it follows from (84) that

(85) $$\begin{array}{c} \varphi_{N}\left(\Delta_{k}\right)\subseteq Y\times \left\{w\in X:\mu_{1}\left(y,w\right)<\alpha \right\},\\ \varphi_{N}\left(\Delta_{k}\right)\subseteq \left\{z\in Y:\mu_{1}\left(z,x\right)>\alpha \right\}\times X. \end{array}$$

Therefore, we have *φ*_*N*_(Δ_*k*_)⊆{(*z*, *w*) ∈ *Y* × *X* : *υ*((*y*, *x*), (*z*, *w*)) > 0}, which implies that for each (*y*, *x*) ∈ *Y* × *X*, the set {(*z*, *w*) ∈ *Y* × *X* : *υ*((*y*, *x*), (*z*, *w*)) > 0} is an *FWC*-subspace of (*Y* × *X*; *φ*_*N*_1__^1^ × *φ*_*N*_2__^2^). Thus, by Corollary 35 with *X* = *D* and *μ*_1_ = *ν*_1_, there exists $(\hat{y},\hat{x})\in \bar{T(Y\times X)}$ such that $\upsilon ((\hat{y},\hat{x}),(z,w))\leq 0$ for each (*z*, *w*) ∈ *Y* × *X*. Hence, for each (*z*, *w*) ∈ *Y* × *X*, either $\mu_{1}(z,\hat{x})\leq \alpha$ or $\mu_{1}(\hat{y},w)\geq \alpha$, which contradicts (80). Therefore, we have inf⁡_*x*∈*X*_sup⁡_*y*∈*Y*_ *μ*_1_(*y*, *x*) = sup⁡_*y*∈*Y*_inf⁡_*x*∈*X*_ *μ*_1_(*y*, *x*). This completes the proof.

**Remark 45**

Theorem 44 generalizes Theorem 4.4 of Tan [41] in the following aspects: (a) The underlying spaces of Theorem 44 are *FWC*-spaces which contain* G-*convex spaces adopted in Theorem 4.4 of Tan [41]; (b) the assumption that each *G*-co⁡(*A*) and each *G*-co⁡(*B*) in Theorem 4.4 of Tan [41] are compact is dropped; (c) (ii) of Theorem 44 is weaker than (1) and (2) of Theorem 4.4 of Tan [41]; (d) (iii) and (iv) of Theorem 44 are weaker than (3) of Theorem 4.4 of Tan [41].
